# Tuning the Emission of Bis-ethylenedioxythiophene-thiophenes upon Aggregation

**DOI:** 10.1021/acs.jpcb.4c02891

**Published:** 2024-06-28

**Authors:** Ihor Sahalianov, Tobias Abrahamsson, Diana Priyadarshini, Abdelrazek H. Mousa, Katriann Arja, Jennifer Y. Gerasimov, Mathieu Linares, Daniel T. Simon, Roger Olsson, Glib Baryshnikov, Magnus Berggren, Chiara Musumeci

**Affiliations:** † Laboratory of Organic Electronics, Department of Science and Technology, Linkoping University, Norrkoping SE-60174, Sweden; ‡ Wallenberg Initiative Materials Science for Sustainability, ITN, Linköping University, Norrköping 60174, Sweden; § Department of Chemistry and Molecular Biology, University of Gothenburg, SE-405 30, Gothenburg, Sweden; ∥ Group of Scientific Visualization, Department of Science and Technology, Linkoping University, Norrkoping SE-60174, Sweden; ⊥ Chemical Biology & Therapeutics, Department of Experimental Medical Science, Lund University, Lund SE-221 84, Sweden

## Abstract

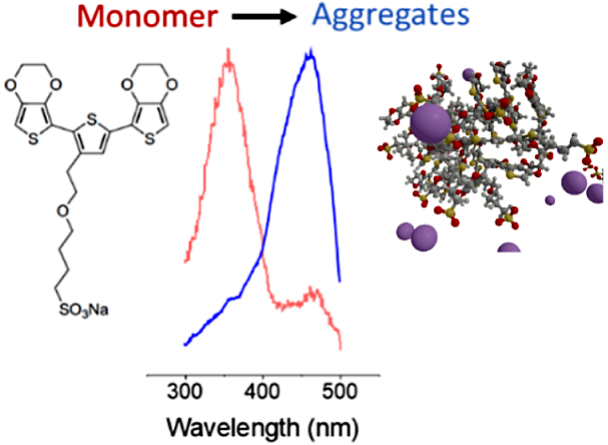

The ability of small lipophilic molecules to penetrate the blood–brain barrier through transmembrane diffusion has enabled researchers to explore new diagnostics and therapies for brain disorders. Until now, therapies targeting the brain have mainly relied on biochemical mechanisms, while electrical treatments such as deep brain stimulation often require invasive procedures. An alternative to implanting deep brain stimulation probes could involve administering small molecule precursors intravenously, capable of crossing the blood–brain barrier, and initiating the formation of conductive polymer networks in the brain through *in vivo* polymerization. This study examines the aggregation behavior of five water-soluble conducting polymer precursors sharing the same conjugate core but differing in side chains, using spectroscopy and various computational chemistry tools. Our findings highlight the significant impact of side chain composition on both aggregation and spectroscopic response.

## I Introduction

The ability of small, lipophilic molecules to pass through the blood–brain barrier by transmembrane diffusion^1^ has allowed researchers to develop new diagnostics^2^ and therapies^3^ for disorders of brain function. However, brain-targeted therapeutics have almost exclusively been based on a biochemical mode of action while electrical therapies have been neglected because they, like deep brain stimulation, often require invasive procedures.^4^ An alternative to implanting deep brain stimulation probes would be to intravenously inject small molecule precursors capable of passing the blood–brain barrier, which can be triggered to produce networks of conductive polymers in the brain via *in vivo* polymerization.

Synthesis of conductive materials directly within living tissue has been achieved in the past. For example, Martin et al. pioneered *in vivo* electropolymerization of electrodes for neural stimulation^5^ and neural prosthesis applications.^6^ A more gentle approach, which takes advantage of endogenous enzymatic activity, has also been applied to polymerize the trimeric thiophene based monomer 2,5-bis(2,3-dihydrothieno[3,4-*b*][1,4]dioxin-5-yl)thiophene (ETE, for “EDOT–thiophene–EDOT”) backbone, functionalized with a single sodium 4-ethoxy-1-butanesulfonate side chain group on the central thiophene unit (ETE-S), within the vasculature of plants^7−10^ and in an invertebrate model, the hydra.^11^ We recently demonstrated enzymatic polymerization fueled by endogenous metabolites for the *in vivo* formation of substrate-free organic electronics, in both vertebrate and invertebrate models.^12^

While ETE materials show great promise in next-generation bioelectronic interfaces, the conjugated thiophene backbone of ETE is hydrophobic by nature, which suppresses the solubility in water. Furthermore, ETEs have a tendency to aggregate in solution through π–π stacking interactions, which could even block their ability to pass through membranes, polymerize, or both. However, the ETE structure can be functionalized with hydrophilic side chains to tune both the solubility and the aggregation properties.

**Figure 1:**
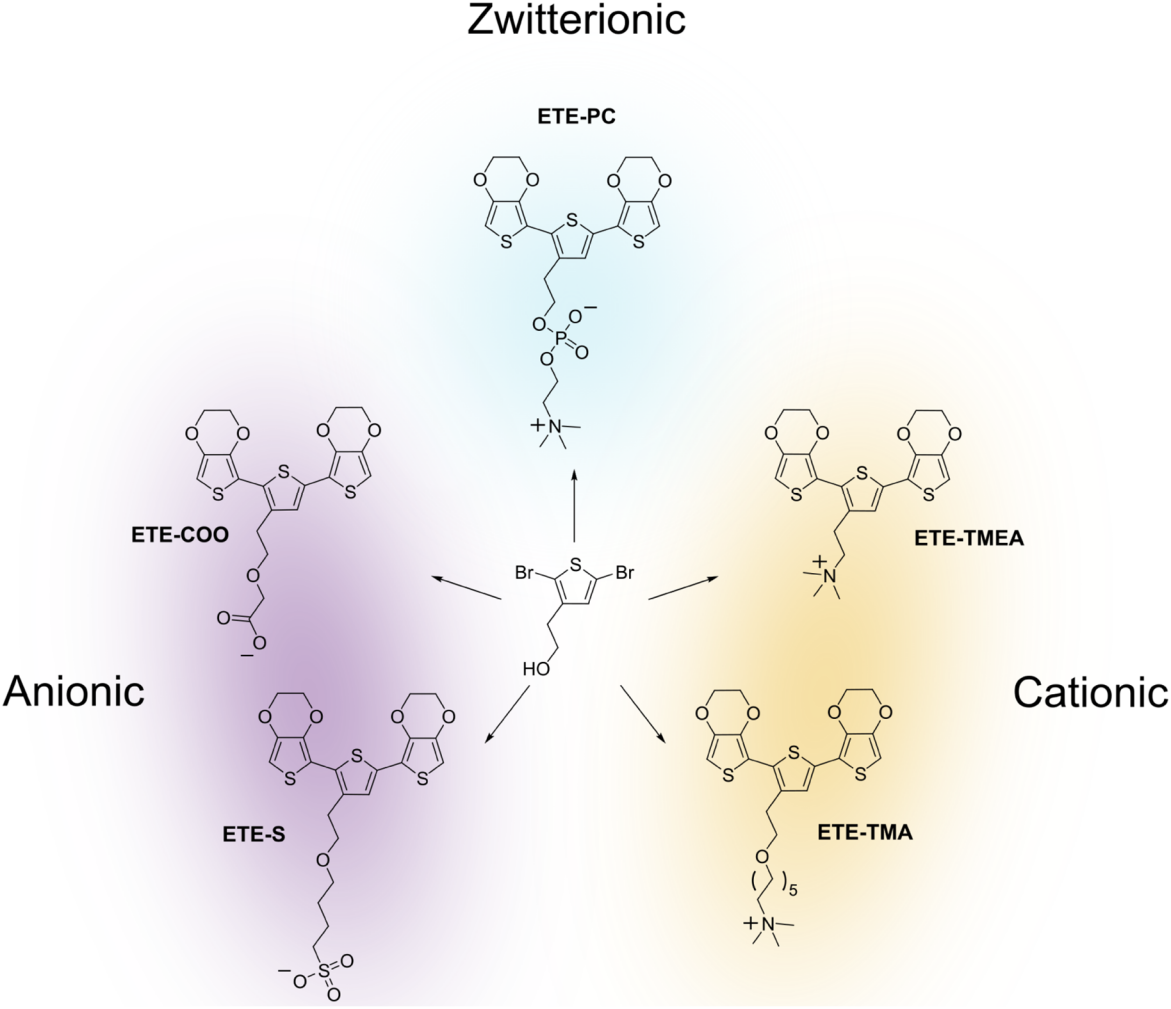
Functionalized ETE (“EDOT–thiophene–EDOT”) monomers and precursor 2-(2,5-dibromothiophen-3- yl)ethanol molecular structures. Anionically charged ETE-COO and ETE-S, cationically charged ETE-TMEA (short alkyl chain) and ETE-TMA (long alkyl chain), and zwitterion/neutral ETE-PC.

In this study, we investigate how the properties of the hydrophilic side chain can affect molecular aggregation and the intermolecular interactions within the aggregates by comparing ETE derivatives bearing an anionic sulfonate (ETE-S) or carboxylate (ETE-COO) side chain group, a zwitterionic phosphocholine group (ETE-PC),^13^ or a cationic trimethylammonium with a short (ETE-TMEA) or long (ETE-TMA) alkyl side chain (Figure 1). We probe the energy transfer between neighboring molecules by UV–vis absorption and fluorescence spectroscopy as a function of the ETE concentration. Spectroscopy results are analyzed with the support of molecular modeling, combining with molecular dynamics (MD) simulation to investigate the aggregation and time-dependent density functional theory (TD-DFT) for the analysis of the nature of the absorption and emission bands upon aggregation.

## II Methods

### II. A Experimental Methods

2-(2,5-Dibromothiophen-3-yl)ethanol, sodium 4-(2-(2,5-bis(2,3-dihydrothieno[3,4-*b*][1,4]dioxin-5-yl)thiophen-3yl)ethoxy)butane-1-sulfonate (ETE-S), 2-(2,5-bis(2,3-dihydrothieno[3,4-*b*][1,4]dioxin-5-yl)thiophen-3-yl)ethyl(2-(trimethylammonio)ethyl) phosphate (ETE-PC), sodium 2-(2-(2,5-bis(2,3-dihydrothieno[3,4-*b*][1,4]dioxin-5-yl)thiophen-3-yl)ethoxy)acetic acid salt (ETE-COO), and 6-(2-(2,5-bis(2,3-dihydrothieno[3,4-*b*][1,4]dioxin-5-yl)thiophen-3-yl)ethoxy)-N,N,*N*-trimethylhexan-1-ammonium bromide (ETE-TMA) were synthesized as previously described.^12−14^ The synthesis and characterization of 2-(2,5-bis(2,3-dihydrothieno[3,4-*b*][1,4]dioxin-5-yl)thiophen-3-yl)-N,N,N-trimethylethanammonium acetate (ETE-TMEA) are reported in Figures S1–S12.

Absorbance and photoluminescence spectra of the ETE derivatives solutions in deionized water at different concentrations (0.02–4 mM) were measured in a Synergy H1 plate reader (BioTek), operating at room temperature.

### II. B Theoretical Methods

Absorption properties of different compounds were investigated with density functional theory atomistic simulations. We used the CAM-B3LYP functional^15^ with D3 Grimme empirical correction^16^ to correctly describe interactions between monomers. All systems underwent geometrical optimization in water implicit solvent (implemented with the polarized continuum model^17^) followed by a frequency check. Absorption spectra were obtained with time-dependent density functional theory^18^ taking into account the first 200 transitions. Simulations were carried out in the Gaussian 16 software package.^19^ All compounds were simulated in 6-31G(d) basis sets.

For simulations of monomer aggregate geometries and radial distribution functions, we used the following procedure. Topologies for the MD simulation were created using the LigParGen server^20−22^ using the DFT calculations and general amber force field (OPLS-AA) parameters. All MD simulations were carried out using the GROMACS program.^23−26^ Boxes were built by placing the molecules in a random position and by solvating them with water molecules using the TIP4Pew water model.^27^ Charged monomers were neutralized by adding ions to the solution. ETE-S and ETE-COO were neutralized using Na^+^ ions while ETE-TMA and ETE-TMEA were neutralized with Cl^–^. For the study of the small aggregates with 20 molecules, the box size was 7 × 7 × 7 nm^3^. The system was equilibrated in the NVT for 10 ns and in the NPT ensemble for 10 ns with a time step of 2 fs. For equilibrations, the temperature of the system was kept constant at 300 K with the V-rescale^28^ modified Berendsen thermostat in both NVT and NPT and Parrinello–Rahman^29^ pressure coupling in NPT. For the production run, the temperature of the system was kept constant at 300 K with a pressure at 1 atm for 100 ns with a step of 2 fs. Visualization of MD data was done with VIAMD software.^30^

## III Results and Discussion

**Figure 2:**
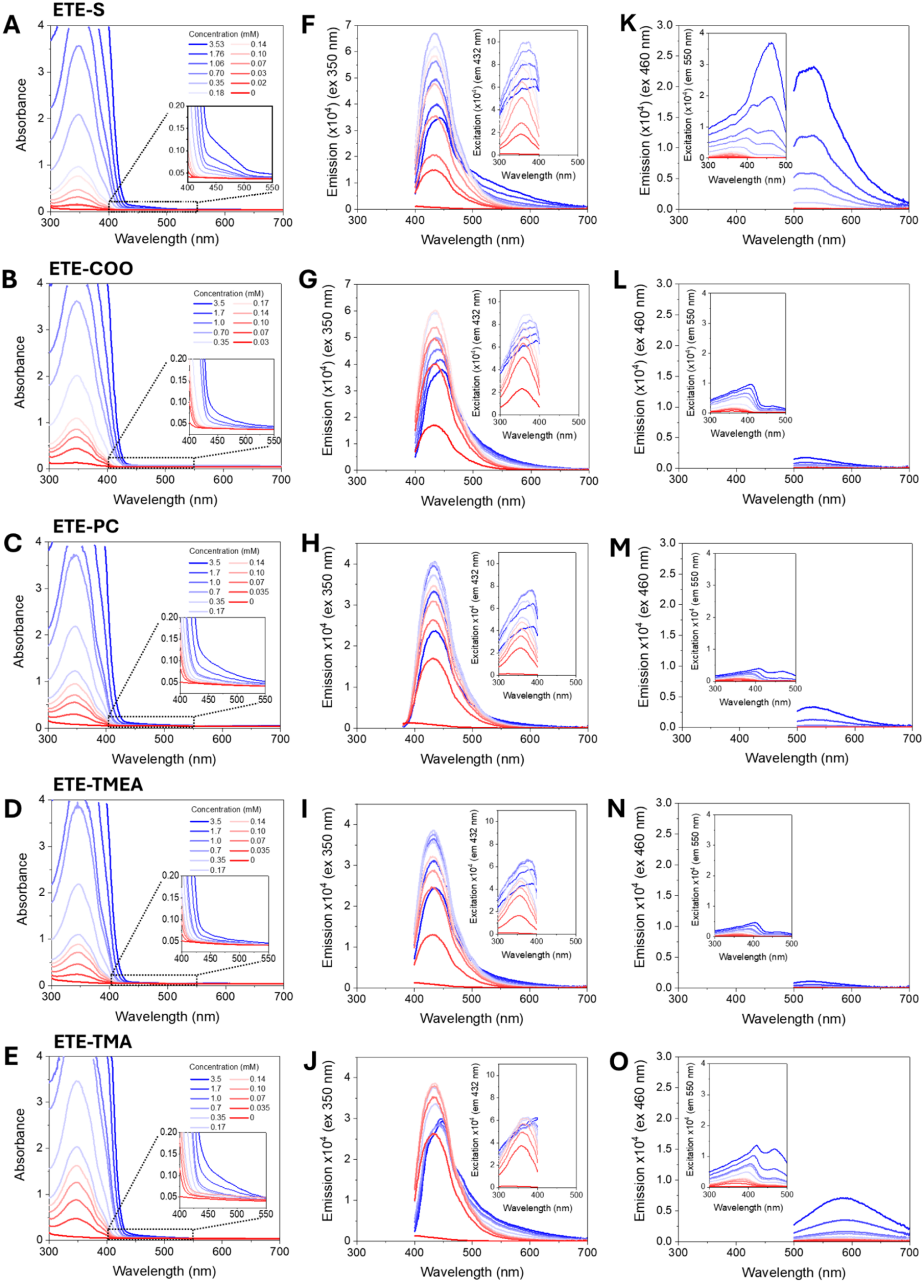
Experimental absorption and emission spectra for ETE-S (A, F, K), ETE-COO (B, G, L), ETE-PC (C, H, M), ETE-TMEA (D, I, N), and ETE-TMA (E, J, O) at different concentrations in water. The insets in Figure 2A–E are enlargements of the absorption spectra in the 400–550 nm range. Emission spectra were measured at excitation wavelengths of 350 nm (F–J) and 460 nm (K–O), and the corresponding excitation spectra at 432 and 550 nm are shown in the insets.

Absorbance spectra of diluted solutions show that the absorption maximum of the monomers is very similar for all the ETEs and it is centered at 345–348 nm. Upon increasing the concentration above 0.3–0.4 mM, we observe the formation of an absorption band at around 460 nm, suggesting aggregation and π–π stacking interaction. This band is relatively more pronounced for ETE-S and ETE-TMA than for the other ETEs (Figure 2A–E).

Upon excitation at 350 nm, which corresponds to the monomeric band, an emission band centered at 434 nm is obtained for all of the molecules. At high concentrations, the intensity of emission decreases, likely due to quenching, and the position of the emission peaks shifts (2–15 nm) toward longer wavelengths (see Figures 2F–J and S13). When excited at 460 nm, an emission band is observed only for high concentrations (Figure 2K–O), which strongly indicates that this band can be attributed to emission from the aggregates. This band is also much more pronounced for ETE-S and ETE-TMA than for ETE-PC, ETE-COO, and ETE-TMEA, which further signifies that those molecules must form different types of aggregates.

The excitation spectra also support the observation that species absorbing at around 350 nm are responsible for the emission at 430–440 nm (insets of Figure 2F–J), but different species, absorbing at 460 nm, are responsible for the emission at higher wavelengths, namely, 550 nm (insets of Figure 2K–O). Interestingly, the excitation peak at 460 nm is 3–4 times smaller for ETE-PC, ETE-COO, and ETE-TMEA compared to ETE-S, again corroborating a difference in aggregation between the different molecules.

Dynamic light scattering (DLS) measurements also seem to confirm the presence of aggregates in concentrated solutions (Figure S14). However, due to complex scattering patterns, orientation effects, and misleading hydrodynamic radius interpretations, which are to be expected when dealing with nonspherical objects, DLS is not suitable to quantitatively describe and compare the aggregation behavior of these systems.

**Figure 3:**
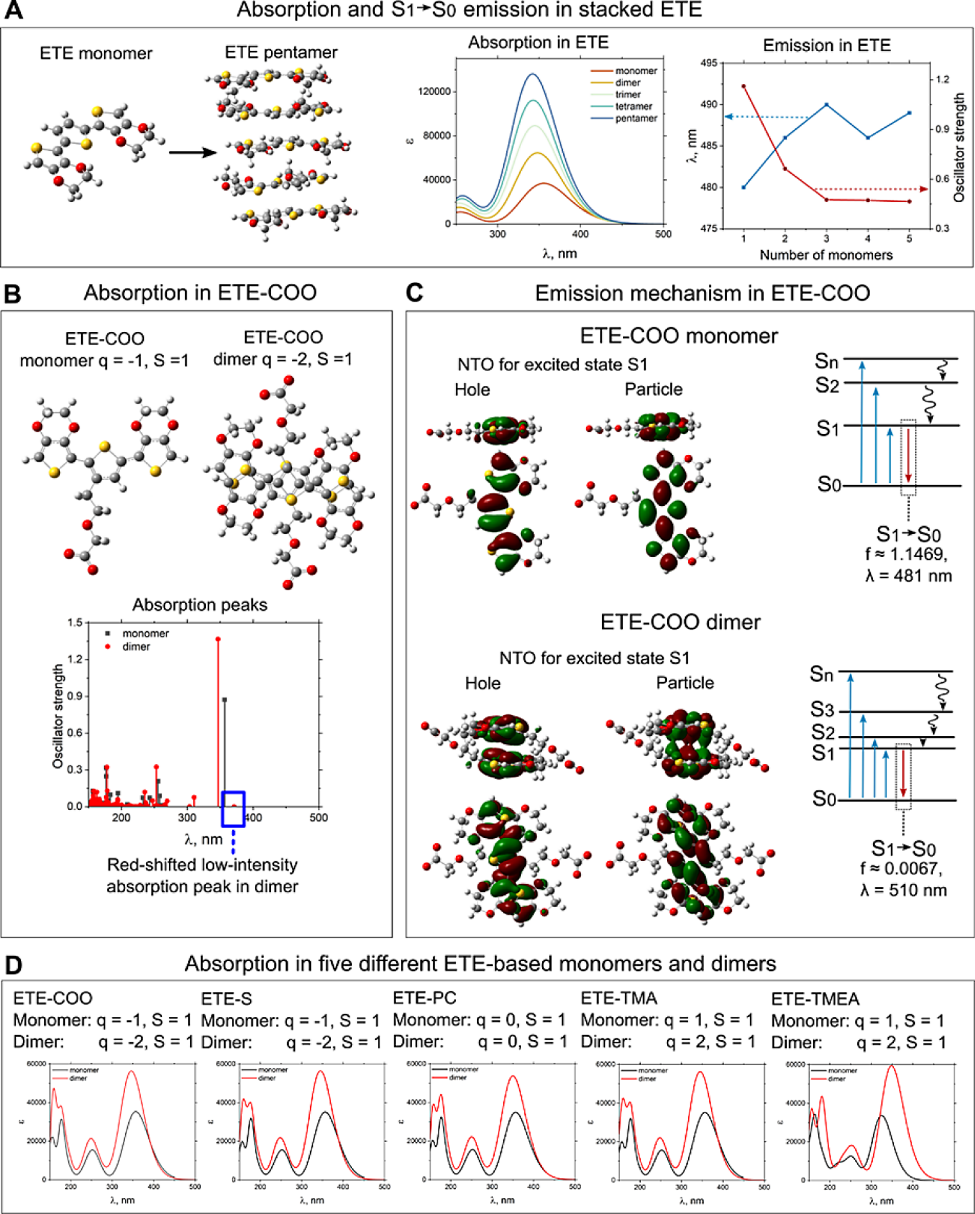
Absorption and emission in stacked ETE depending on the number of monomers in the crystallite. (A) Simulated geometry and absorption properties in monomer and dimer ETE-COO. (B) Absorption peaks were convolved with the peak smearing half-width half-maximum 0.33 eV. (C) Natural transition orbitals simulated for the excited state S_1_ of the monomer and dimer ETE-COO. Emission mechanisms for both systems schematically show the details of the S_1_ → S_0_ emissive transition. (D) Absorption of all five ETE-based compounds in both monomer and dimer geometries.

The experimentally recorded changes in emission spectra originate from changes in the electronic structures of monomers upon aggregation. We used time-dependent density functional theory to calculate the absorption and emission of monomers and a π–π-stacked dimer of all five ETE-based compounds. The results are summarized in Figure 3 with a detailed description of S_0_ → S_1_, S_1_ → S_0_ transitions in Tables S1,2. Ground state geometries of ETE-based monomers and dimers are presented in Figure S15.

All five compounds are based on the same ETE core, which is predominantly responsible for the optical properties. We started the studies of the correlation between the aggregation and changes in the absorption and emission proportion by simulating stacked ETE aggregates from monomer up to pentamer form (Figure 3A). With an increase in the number of monomers in an aggregate, the absorption spectra undergo an increase in amplitude and slight broadening. There is a minor blue-shift in the main absorption peak located near λ ≈ 350 nm. The emission properties were studied by calculating the S_1_ → S_0_ transition (Figure 3A). We found that an increase in the number of monomers in the aggregate leads to the emission at a longer wavelength than in the case of individual ETE monomers. Also, there is a correlation between the number of monomers in a stack and the oscillator strength value. We observed a substantial decrease in oscillator strength from 1.16 to 0.47, reaching a plateau at three monomers in the aggregate. This result can be associated with aggregation-induced quenching of emission. According to Strickler–Berg equation, the radiative rate constant is directly proportional to the oscillator strength and energy of the transition^31,32^
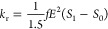
where *f* is the oscillator strength and *E* (cm^–1^) is the energy of S_1_ → S_0_ emissive transition. Both the emission red-shift and the decrease in oscillator strength lead to the lower radiative rate constant. However, the dominant contribution to quenching originates from the decrease in oscillator strength because of a small decrease in energy of the S_1_ → S_0_ transition.

Figure3B, C presents the absorption and emission properties of the negatively charged ETE-COO monomer and dimer as representative systems. For the monomer, the first absorption band is centered at 357 nm and for the dimer at 347 nm, which is in good agreement with the experiment. In the spectrum of the dimer compound, π–π stacking results in the appearance of another small intensity absorption peak, located at 371 nm (Figure 3B). Even though this peak is not apparent in the experimental absorption spectra, it significantly affects the emission properties of the compound because of the small oscillator strength. Transition S_1_ → S_0_ occurs at lower energy than absorption S_0_ → S_1_, which results in quenched red-shifted emission (Tables S1 and S2). By looking at the natural transition orbitals (NTOs, Figure 3C), we can conclude that both hole and particle visualizations are delocalized over the whole conjugated core of the ETE. In the case of a dimer compound, a hole is delocalized over the ETE-core monomers. Particle visualization shows bonding interaction between ETE cores, which suggests that the stacked structure will be even more stable in an excited state. After optimization of the molecule at the first excited state (S_1_), we observe the ETE-COO anion emission, originating from the S_1_ → S_0_ transition at λ = 481 nm. However, the π–π interaction in the dimer results in the S_1_ → S_0_ transition at a slightly higher wavelength (510 nm) but substantially lower oscillator strength. This fully agrees with the emission redshift and quenching of emission at high ETE-COO concentrations reported experimentally. Similar aggregation-induced absorption changes were observed for other neutral and charged compounds, except ETE-TMEA, where absorption spectra of the dimer were red-shifted compared to those of the monomer (Figure 3D).

**Figure 4:**
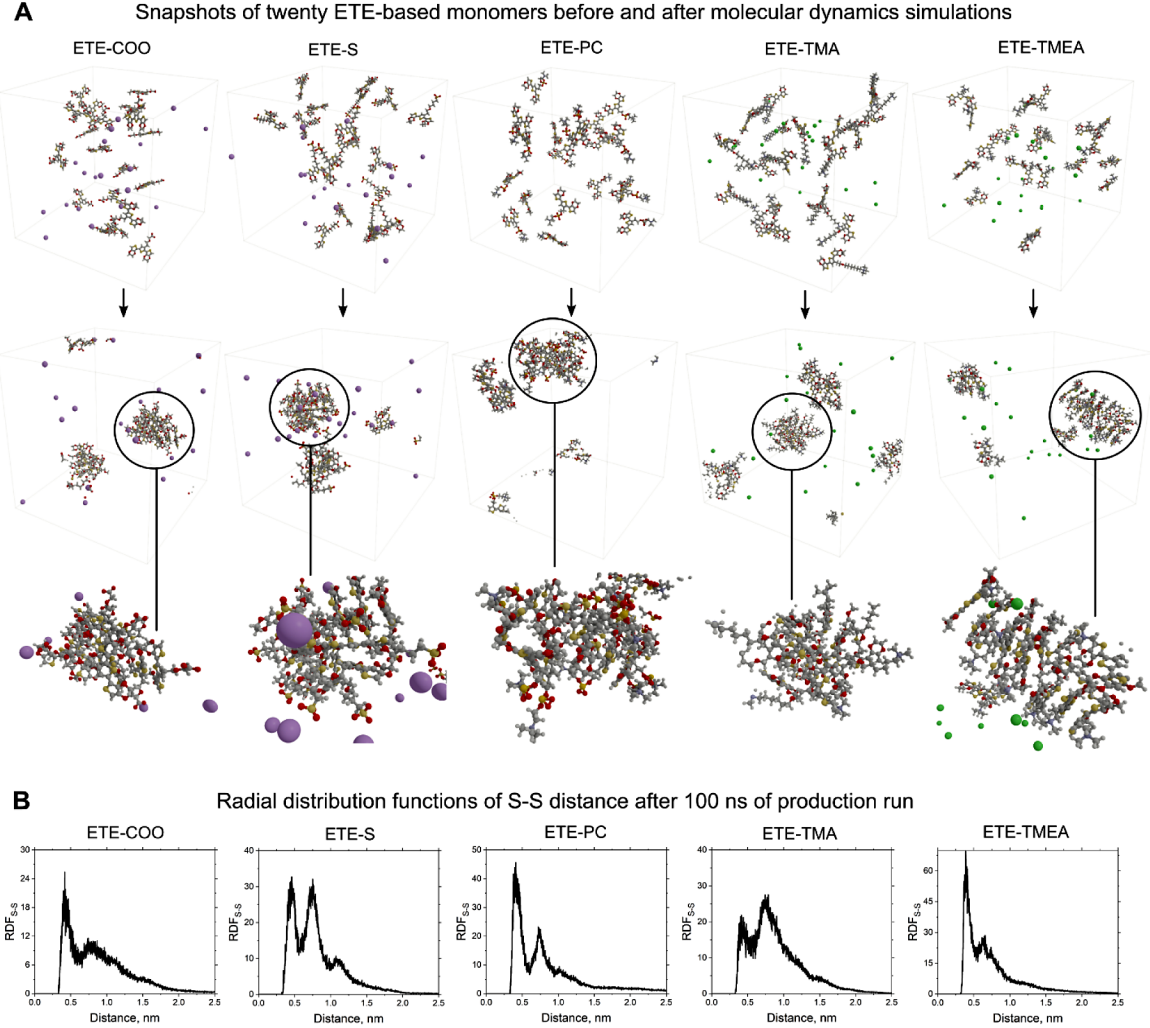
Molecular dynamics simulations of five different ETE compounds. Predicted aggregation of monomers into clusters of aggregates after equilibration and 100 ns of a production run. (A) Calculated S–S radial distribution functions of the sulfur atom in the central thiophene ring of ETE-based monomers (B).

The aggregation behavior of ETE-based compounds was analyzed by molecular dynamics studies. For each of the compounds, we created a simulation box, filled with 20 monomers (balanced with counterions, if necessary), and solvated in water. The size of the box was chosen to be 7 × 7 × 7 nm^3^ to allow uniform initial spatial distribution of monomers and free movement during the equilibration (Figure 4A) at room temperature. After equilibration and a 100 ns production run, the ETE-based monomers aggregated into clusters. Aggregates visually resemble semiamorphous clusters (ETE-COO) or more ordered stacks (ETE-S or ETE-TMEA). In the case of charged ETE compounds, counterions remained at a distance from the backbone and the side chains and did not diffuse into the aggregates (Figure 4A).

For a better understanding of the aggregates’ structure, we calculated the radial distribution functions (RDFs) between the ETE-based monomers (Figure 4B). Because of a large difference in the side chain structure, the positions of the center of mass vary significantly. To mitigate this issue and potential distortion in results, we calculate the RDF between the sulfur atoms in the central thiophene of the ETE, which represents the π–π stacking between ETE cores. RDF for all five compounds shows a sharp peak corresponding to the first coordination shell stacking (Table S3). The value of the intermonomer distance at the first coordination shell is the largest for ETE-S (0.45 nm) and the smallest for ETE-TMEA (0.38 nm). The second coordination peak is present in all RDF graphs at approximately the same position of ≈0.75 nm (Figure 4B and Table S3). The compounds ETE-S and ETE-TMA have comparable amplitudes of the first and second peaks. We believe it is a consequence of their long and charged side chains. In this case, there is a strong tendency to order in the second coordination shell with side chains participating in aggregation in the first coordination shell. As for the third coordination shell, only ETE-S shows a clear third peak in RDF, thus having more elongated crystallites in aggregates compared with other compounds. The general lack of a third peak and smooth plateau in RDFs suggests a semiamorphous structure of aggregates. Comparing the values of RDF for different compounds, we can conclude that the probability of the second-shell arrangement is roughly the same for all compounds except ETE-COO. The probability of the arrangement in the first coordination shell is the largest for ETE-TMEA and ETE-PC.

The MD studies conclude that all compounds form aggregates spontaneously with a particular degree of short-range order. The most ordered aggregate was formed by ETE-S and ETE-TMEA, while the most amorphous aggregate (still with some dimer and trimer stacking) was formed by ETE-COO. In three of four compounds, charged side chains additionally stimulate aggregation in the second coordination shell. However, long side chains interfere with π–π stacking at the first coordination shell. Even though minor changes in RDF might be possible in the case of simulations of larger systems consisting of more monomers, the hypothesis of aggregation was verified and confirmed.

Signatures of aggregation are observed in the absorption spectra (Figure 2). With an increase of the monomer concentration from 0.03 to 3.5 mM, a shoulder in the range 400–550 nm appears. The intensity of this absorption grows proportionally to the increase of the monomer concentration and varies depending on the type of side chain. The band at 400–550 nm is most intensive for ETE-S, less intensive for ETE-PC and ETE-TMA, and the smallest for ETE-COO and ETE-TMEA (Figure 2). These experimental observations are consistent with the molecular dynamics simulations. As shown in Figure 4, MD simulations show an orderly stacked structure for the ETE-S aggregates. Compared with other ETE compounds, the radial distribution function of ETE-S contains a well-defined third peak, corresponding to the interaction in the third solvation sphere. Also, MD simulations predict a more amorphous aggregate of ETE-COO, with low-intensity first and second RDF peaks, which is in agreement with the experimentally recorded low-intensity absorption shoulder for this compound (Figure 2). The only case of a slight misalignment between theory and experiment is ETE-TMEA. The experiment shows the formation of aggregates of ETE-TMEA (Figure 2). However, the intensity of absorption at 400–550 nm is smaller than in other compounds. While MD confirmed the aggregation of ETE-TMEA, it also predicts that its aggregates should exhibit more order, considering the large intensity of its RDF. This is a consequence of the restrictions of simulations, which were carried out at larger concentrations of monomers than concentrations used in the experiment. It seems that at smaller concentrations (<3.5 mM), longer side chains play an essential role in aggregate formation, which is proven by the larger absorption at 400–500 nm for ETE-S and ETE-TMA.

## IV Conclusion

In summary, we investigated the aggregation of five water-soluble conducting polymer precursors, ETE-S, ETE-COO, ETE-PC, ETE-TMEA, and ETE-TMA, aiming to understand the short-range structure and molecular interactions within these aggregates. Using TD-DFT calculations, we identified the nature of the absorbance and emission spectra, revealing that the π–π interaction in the dimer resulted in a S1 → S0 transition at a slightly longer wavelength but substantially lower oscillator strength than the monomer. This finding correlated well with the experimentally observed emission red-shift and quenching at high concentrations, serving as distinctive indicators of aggregation in solution. This shifted emissive band can be used in the future to screen for the aggregation of novel ETE-based molecules. Despite sharing an identical core, ETE derivatives exhibited varying degrees of order in their aggregated forms, as confirmed by molecular dynamics simulations. This diversity was influenced not only by the charge of the side group but also by the distance of the charged group from the core. The rich and descriptive information obtained from the combination of experiment, calculation, and modeling is extremely valuable when designing and real-time monitoring the self-assembly processes of conducting polymer precursors in aqueous systems like those required for *in vivo* polymerization.

## Supporting Information Available

ETE-TMEA, additional spectroscopy data, dynamic light scattering, and additional modeling data (PDF)
