# Supplementary material for: Tuning the Emission of Bis-ethylenedioxythiophene-thiophenes upon Aggregation
Source: J Phys Chem B. 2024 Jun 28;128(27):6581–8. doi: 10.1021/acs.jpcb.4c02891 (PMC11247477; doi:10.1021/acs.jpcb.4c02891)
Supplement: Supplementary file 1 — jp4c02891_si_001.pdf [file jp4c02891_si_001.pdf]

Supporting Information

Tuning the Emission of Bis-Ethylenedioxythiophene-Thiophenes upon Aggregation

Ihor Sahalianov,[a, f, §] *Tobias Abrahamsson,*[a §] *Diana Priyadarshini,*[a] *Abdelrazek H. Mousa,*[c] *Katriann Arja,*[a] *Jennifer Gerasimov,*[a] *Mathieu Linares,*[a,b] *Daniel T. Simon,*[a] *Roger Olsson,*[c,d,] *Glib Baryshnikov,*[a,e*] *Magnus Berggren*[a] *Chiara Musumeci,*[a, *]

[a]Laboratory of Organic Electronics, Department of Science and Technology, Linkoping University, SE-60174, Norrkoping, Sweden.

[b]Group of Scientific Visualization, Department of Science and Technology, Linkoping University, SE-60174, Norrkoping, Sweden.

[c]Department of Chemistry and Molecular Biology, University of Gothenburg, SE-405 30, Gothenburg, Sweden.

[d]Chemical Biology & Therapeutics, Department of Experimental Medical Science, Lund University, SE-221 84, Lund, Sweden.

[e]Wallenberg Initiative Materials Science for Sustainability, ITN, Linköping University, 60174 Norrköping, Sweden

[§] These authors contributed equally.

*Glib Baryshnikov: Email: glib.baryshnikov@liu.se

*Chiara Musumeci. Email: chiara.musumeci@liu.se

- Synthetic Methods
- Synthesis and characterization of ETE-TMEA
- Additional spectroscopy data
- Dynamic Light Scattering (DLS)
- Additional modelling data
- References

Synthetic Methods

The commercial chemicals used in the synthesis were used as received without further purification. Synthesis and characterization of the monomers: 2-(2,5-dibromothiophen-3-yl)ethanol, 2,3-dihydrothieno[3,4-b][1,4]dioxin-5-yl)-4,4,5,5- tetramethyl-1,3,2-dioxaborolane, sodium 4-(2-(2,5-bis(2,3- dihydrothieno[3,4-b][1,4]dioxin-5-yl)thiophen-3-yl)ethoxy)butane-1-sulfonate (ETE-S), 2-(2,5-bis(2,3-dihydrothieno[3,4-b][1,4]dioxin-5-yl)thiophen-3-yl)ethyl(2-(trimethylammonio)ethyl) phosphate (ETE-PC), sodium 2-(2,5-bis(2,3-dihydrothieno[3,4-b][1,4]dioxin-5-yl)thiophen-3-yl)ethoxy)acetic acid salt (ETE-COO) and 6-(2-(2,5-bis(2,3-dihydrothieno[3,4-b][1,4]dioxin-5-yl)thiophen-3-yl)ethoxy)-N,N,N- trimethylhexan-1-aminium bromide (ETE-TMA) have previously been reported.[1-3] ^{1}H- and ^{13}C-NMR were performed with an Inova Varian Oxford AS500 spectrometer, recorded at 500 and 126 MHz respectively. Chemicals shifts were referenced against solvent residual peaks of chloroform-*d*, acetone-d_6 and methanol-d_4.[4] MestReNova 12.04 (Mestrlab Research) was used for NMR analysis and processing. High performance liquid chromatography-mass spectrometry (HPLC-MS) analysis was performed on a Waters system equipped with a 2767 Sample Manager, 2 x 515 HPLC pump, 2424 Evaporative Light Scattering (ELS) Detector, 2998 Photodiode Array (PDA) Detector, SQ Detector 2 (single quadrupole mass detector, ESI ionization), XBridge BEH C18 Column (3.5 µm x 4.6 mm x 50 mm, pore size 130 Å). Mobile water phase A (95 % H_2O, acetonitrile 5 %, 10 mM NH_4OAc) and organic phase B (10 % H_2O, 90% acetonitrile, 10 mM NH_4OAc) were used in 30-100% or a 10-100% (organic phase B) gradient (4 CV), 1.5 mL/min, injection volume 20 µL.

Synthesis and characterization of ETE-TMEA

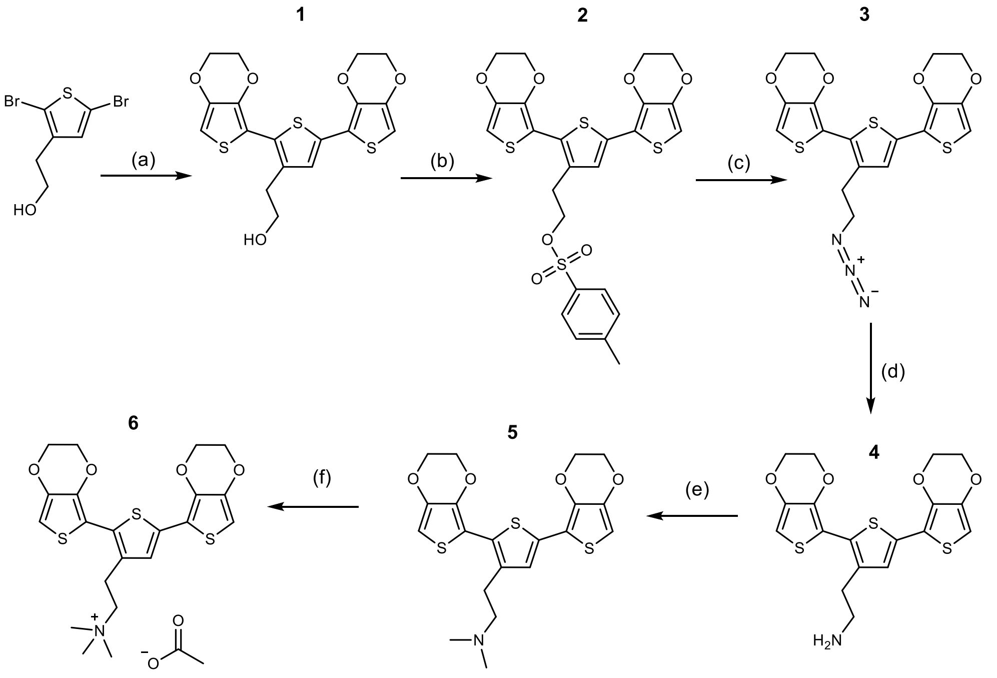

Scheme S1. Synthesis of ETE-TMEA (**6**). (a) 2,3-dihydrothieno[3,4-b][1,4]dioxin-5-yl)-4,4,5,5-tetramethyl-1,3,2-dioxaborolane (2.10 eq.), PEPPSI-iPr (5 mol %), 1 M Na_2CO_3 (aq.) (2.09 eq.) in THF, 80 °C, 21h, 91%. (b) 4-methylbenzenesulfonyl chloride (3 eq.) in pyridine, 16h, 40 °C, 26 %. (c) NaN_3 (3 eq.) in DMF, 80 °C, 16h, 81 %. (d) PPh_3 (1.1 eq.) in THF, 50 °C, 1 M NaOH (aq.) (8.82 eq.), 21h, 49 %. (e) Glacial acetic acid (3.95 eq.), sodium cyanoborohydride (2.64 eq.), formaldehyde solution (37 %, aq.) (10 % methanol, 3.00 eq.) in methanol, 18h, formaldehyde solution (37 %, aq.) (10 % methanol, 3.00 eq.), 3h, 54 %. (f) Potassium carbonate (3.15 eq.), iodomethane (225 eq.), reflux, 2½ h, ammonium acetate (aq.), 58 %.

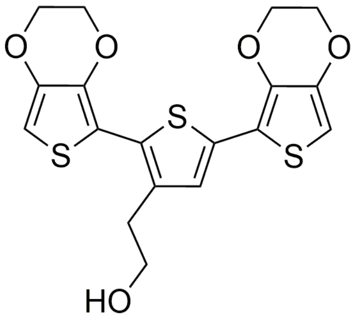

2,5-Bis(2,3-dihydrothieno[3,4-*b*]-1,4-dioxin-5-yl)-3-thiopheneethanol (**1**)

In a round bottom flask were 2,5-dibromo-3-thiopheneethanol (2424 mg, 8.476 mmol), 2,3-dihydrothieno[3,4-b][1,4]dioxin-5-yl)-4,4,5,5- tetramethyl-1,3,2-dioxaborolane (6041 mg, 17.799 mmol, 2.1 eq.) and PEPPSI-iPr (5 mol %, 288 mg, 0.424 mmol) dissolved in THF (85 mL). To the solution was 1 M Na_2CO_3 (17.7 mL, 2.09 eq.) added and nitrogen was bubbled for 15 min. The reaction was heated to 80 ^{o}C and left to stir for 21h. After reaching room temperature was the solution filtered through a small silica gel plug (with additional THF wash). The crude product was dry loaded onto silica via rotary evaporation and applied on a 50 g silica column. By using a heptane:ethyl acetate gradient was the product isolated and after evaporating the solvents was the product left to dry under high vacuum to yield a clear brown oil (3160 mg, 91 %). ^{1}H NMR (500 MHz, Chloroform-*d*) δ 7.10 (s, 1H), 6.36 (s, 1H), 6.21 (s, 1H), 4.34 – 4.30 (m, 2H), 4.28 – 4.25 (m, 2H), 4.25 – 4.21 (m, 4H), 3.86 (s, 2H), 2.94 (t, J = 6.6 Hz, 2H). ^{13}C NMR (126 MHz, Chloroform-*d*) δ 141.93, 141.63, 138.37, 137.80, 136.80, 134.74, 127.36, 125.02, 112.03, 109.82, 99.45, 97.25, 65.11, 65.01, 64.69, 64.57, 62.86, 32.83. MS (ESI) m/z: $[M+H]^+$: 409.302, $[M - H]^-$: 407.271; calculated for $C_{18}H_{16}O_5S_3$: 408.50. UV (PDA): λ_{max} (H_2O/acetonitrile): 351.096 nm.

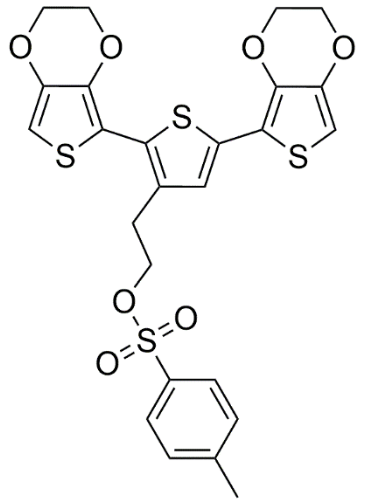

2-(2,5-bis(2,3-dihydrothieno[3,4-*b*]-1,4-dioxin-5-yl)thiophene-3-yl)ethyl 4-methylbenzenesulfonate (**2**)

To a solution of 2,5-bis(2,3-dihydrothieno[3,4-*b*]-1,4-dioxin-5-yl)-3-thiopheneethanol (**1**) (2568 mg, 6.286 mmol) in pyridine (50 mL) cooled with an ice bath was a solution of 4-methylbenzenesulfonyl chloride (3595 mg, 18.857 mmol, 3 eq.) in pyridine (50 mL) added drop wise. The reaction mixture was purged with nitrogen gas and left to stir for 16h at 40 ^{o}C while kept in the dark. Via rotary evaporation was the majority of the pyridine solvent removed and the remains were dissolved in a small amount of dichloromethane and applied onto a 10 g silica plug. The silica plug was assembled onto a 50 g Silica Sfär column and the product was isolated via a heptane:ethyl acetate mobile phase gradient. Product fractions were pooled, the solvents were evaporated and the product was left to dry under high vacuum to yield a sticky brown-yellow solid (929 mg, 26 %). ^{1}H NMR (500 MHz, Chloroform-*d*) δ 7.68 (d, *J* = 8.2 Hz, 2H), 7.24 (d, *J* = 6.0 Hz, 2H), 6.89 (s, 1H), 6.33 (s, 1H), 6.21 (s, 1H), 4.31 (dd, *J* = 5.1, 2.8 Hz, 2H), 4.28 – 4.13 (m, 8H), 2.99 (t, *J* = 7.3 Hz, 2H), 2.37 (s, 3H).^{13}C NMR (126 MHz, Chloroform-*d*) δ 144.71, 141.94, 141.70, 138.49, 137.85, 134.83, 134.03, 133.03, 129.87, 128.02, 127.94, 124.87, 111.83, 109.18,

99.66, 97.32, 69.90, 65.12, 64.94, 64.69, 64.57, 29.15, 21.71. MS (ESI) m/z: $[M+H]^+$: 563.584; calculated for $C_{25}H_{22}O_7S_4$: 562.68. UV (PDA): λ_{max} (H_2O/acetonitrile): 350.236 nm.

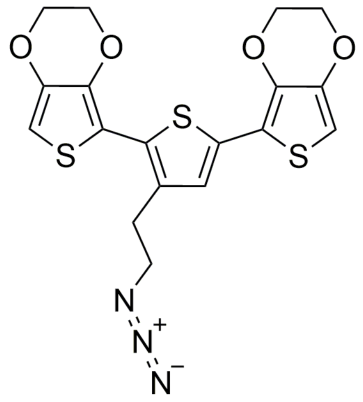

3-(2-azidoethyl) 2,5-bis(2,3-dihydrothieno[3,4-*b*]-1,4-dioxin-5-yl)thiophene (**3**)

Sodium azide (68 mg, 1.046 mmol, 3 eq.) and 2-(2,5-bis(2,3-dihydrothieno[3,4-*b*]-1,4-dioxin-5-yl)thiophene-3-yl)ethyl 4-methylbenzenesulfonate (**2**) (188 mg, 0.334 mmol) were dissolved in DMF (1.5 mL) and the solution was flushed with nitrogen gas for 15 min. The solution was left to stir for 16h at 80 ^oC. After reaching room temperature was water (5 mL) added and the mixture was extracted with ethyl acetate (3x5 ml). The combined organic phases were washed with water (2x15 mL), brine (15 mL), dried with $MgSO_4$ and filtered. The solvents were evaporated and the crude product was dry loaded onto 1 g silica. By applying the silica on a 12g Silica Sfär column was the product isolated with a heptane:ethyl acetate gradient. Solvents were removed via rotary evaporation and the product was left to dry under high vacuum (118 mg, 81%). ^1H NMR (500 MHz, Chloroform-*d*) δ 7.05 (s, 1H), 6.37 (s, 1H), 6.21 (s, 1H), 4.37 – 4.30 (m, 2H), 4.27 (dd, J = 5.9, 2.4 Hz, 2H), 4.23 (dd, J = 5.4, 2.8 Hz, 4H), 3.48 (t, 2H), 2.97 (t, 2H). ^{13}C NMR (126 MHz, Chloroform-*d*) δ 141.95, 141.72, 138.38, 137.86, 135.70, 134.81, 127.65, 124.80, 111.90, 109.63, 99.53, 97.28, 65.11, 64.98, 64.68, 64.58, 51.45, 29.16. MS (ESI) m/z: $[M+H]^+$: 434.426; calculated for $C_{18}H_{15}N_3O_4S_3$: 433.52. UV (PDA): λ_{max} (H_2O/acetonitrile): 350.605 nm.

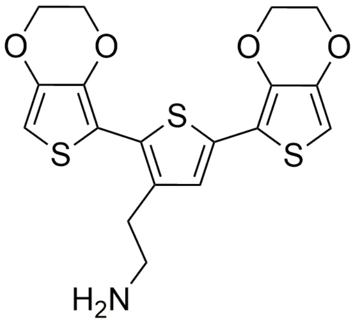

2-(2,5-bis(2,3- dihydrothieno[3,4-b][1,4]dioxin-5-yl)thiophen-3-yl)ethanamine (**4**)

3-(2-azidoethyl) 2,5-bis(2,3-dihydrothieno[3,4-*b*]-1,4-dioxin-5-yl)thiophene (**3**) (118 mg, 0.272 mmol) and triphenylphosphine (79 mg, 0.299 mmol, 1.1 eq.) were dissolved in THF (2.4 mL) and stirred at 50 ^{o}C. Followed by drop wise addition of 1 M NaOH (2.4 mL) and the reaction was left to stir for 21h under nitrogen gas atmosphere. The THF was removed via rotary evaporation and the solution was diluted with water (60 mL) while the pH was adjusted < 3 with 1 M HCl (aq.). The water phase was washed with dichloromethane (3 x 25 mL) and the combined organic phases were extracted additionally with 0.15 M HCl (aq.) (30 mL). All the aqueous phases were combined, the pH adjusted > 8 with 1 M NaOH (aq.) (25 mL) (~100 mL total volume) and extracted with dichloromethane (2x25 mL). The combined organic phases were washed with 1 M NaOH (25 mL), dried with $MgSO_4$ and filtered. The solvent was evaporated and the crude product was dissolved in minimal amounts of methanol and applied on a 12 g C18 silica column. With a water:acetonitrile gradient was the product isolated, the solvents were evaporated and the product was left to dry under high vacuum (54 mg, 49%). ^{1}H NMR (500 MHz, Methanol-d_4) δ 7.12 (s, 1H), 6.51 (s, 1H), 6.34 (s, 1H), 4.36 – 4.32 (m, 2H), 4.29 (dd, *J* = 5.4, 2.7 Hz, 2H), 4.24 (dq, *J* = 6.5, 4.0, 2.9 Hz, 4H), 3.16 (dd, *J* = 9.0, 6.7 Hz, 2H), 3.07 – 2.96 (m, 2H). ^{13}C NMR (126 MHz, Methanol-d_4) δ 143.51,

143.37, 140.04, 139.52, 136.79, 135.27, 129.23, 124.78, 112.30, 109.64, 100.76, 98.31, 66.43, 66.27, 65.84, 65.71, 40.81, 28.45. (ESI) m/z: $[M+H]^+$: 408.316; calculated for $C_{18}H_{17}NO_4S_3$: 407.52. UV (PDA): λ_{max} (H_2O/acetonitrile): 350.453 nm.

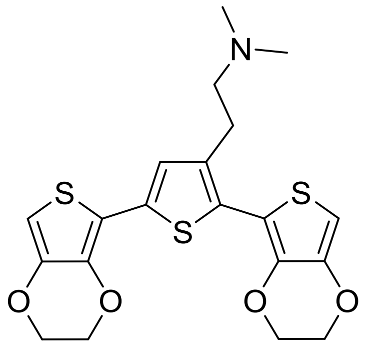

2-(2,5-bis(2,3- dihydrothieno[3,4-b][1,4]dioxin-5-yl)thiophen-3-yl)-N,N-dimethylethanamine (**5**)

A solution of 2-(2,5-bis(2,3- dihydrothieno[3,4-b][1,4]dioxin-5-yl)thiophen-3-yl)ethanamine (**4**) (54 mg, 0.133 mmol) in methanol (2 mL) and glacial acetic acid (30 uL, 0.524 mmol, 3.95 eq.) was added to a micro vial containing sodium cyanoborohydride (22 mg, 0.350 mmol, 2.64 eq.). Addition of 37 % formaldehyde solution (aq., 10 % methanol, 30 uL, 0.399 mmol, 3.00 eq.) to the reaction mixture resulting in immediate hydrogen gas evolution. The reaction was kept in dark and left to stir for 18h. Additional 37 % formaldehyde solution (aq., 10 % methanol, 30 uL, 0.399 mmol, 3.00 eq.) was added and left to stir for 3h. The solvents were reduced via evaporation, the crude residue was loaded onto a C18 silica (6 g) column, and the product was isolated with a water/acetonitrile gradient and isocratic (70/30 → 0/100) mobile phase. Product fractions were pooled, and the solvents were co-evaporated with acetonitrile, yielding a brown oil after drying on high vacuum (31 mg, 54 %). ^{1}H NMR (500 MHz, Methanol-d_4) δ 7.17 (s, 1H), 6.52 (s, 1H), 6.34

(s, 1H), 4.33 (ddd, J = 9.3, 5.5, 3.4 Hz, 4H), 4.28 – 4.21 (m, 4H), 3.37 (dd, J = 9.7, 6.7 Hz, 2H), 3.09 (dd, J = 9.8, 6.6 Hz, 2H), 2.91 (s, 6H). ^{13}C NMR (126 MHz, Methanol-d_4) δ 143.48, 143.42, 140.02, 139.57, 136.94, 134.33, 129.32, 124.80, 112.20, 109.55, 100.91, 98.40, 66.43, 66.35, 65.83, 65.75, 58.64, 43.66, 25.79. HPLC-MS (m/z): calculated for $C_{20}H_{22}NO_4S_3^+$ (436.58); [M + H]$^+$ detected: 436.40. λ_{max} (H_2O/acetonitrile): 350.08 nm.

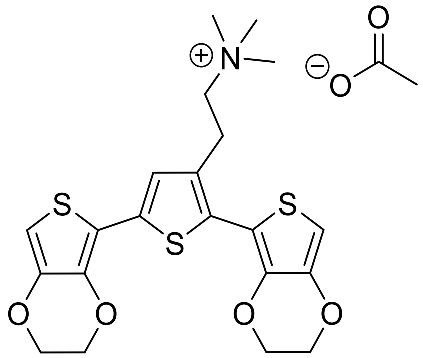

2-(2,5-bis(2,3- dihydrothieno[3,4-b][1,4]dioxin-5-yl)thiophen-3-yl)-N,N,N-trimethylethanaminium acetate (**ETE-TMEA)** (**6**)

To a micro vial containing a solution of 2-(2,5-bis(2,3- dihydrothieno[3,4-b][1,4]dioxin-5-yl)thiophen-3-yl)-N,N-dimethylethanamine (**5**) (31 mg, 0.071 mmol) in THF (1 mL) was potassium carbonate (31 mg, 3.15 eq.) and iodomethane (1 mL, 0.016 mol) added. The vial was caped, and the reaction solution was refluxed for 2½ h. The reaction solution was allowed to reach room temperature, the solvents evaporated, and the crude was dissolved in a minimal amount of water/acetonitrile, followed by wet loading onto a C18 silica (6 g) column. Impurities were removed with a water/acetonitrile mobile phase gradient and the product was elated from the column using 0.01 M ammonium acetate in water/acetonitrile (5:95). Solvents were removed via evaporation and the product was freeze dried thrice to yield a light beige solid (21 mg, 58 %). ^{1}H NMR (500 MHz, Methanol-d_4) δ 7.18 (s, 1H), 6.53 (s, 1H), 6.35 (s, 1H), 4.33 (td, J = 6.2, 2.7 Hz,

4H), 4.28 – 4.22 (m, 4H), 3.66 – 3.42 (m, 2H), 3.19 (s, 9H), 3.06 (s, 2H), 1.89 (s, 3H). ^{13}C NMR (126 MHz, Methanol-d_4) δ 180.15, 143.50, 143.47, 139.95, 139.60, 136.89, 133.64, 129.54, 124.96, 112.16, 109.63, 100.87, 98.44, 67.09, 66.45, 66.41, 65.83, 65.76, 53.69, 53.66, 53.63, 24.33, 24.18. HPLC-MS (m/z): calculated for $C_{21}H_{24}NO_4S_3^+$ (450.61); $[M]^+$ detected: 450.56. λ_{max} (H_2O/acetonitrile): 350.72 nm.

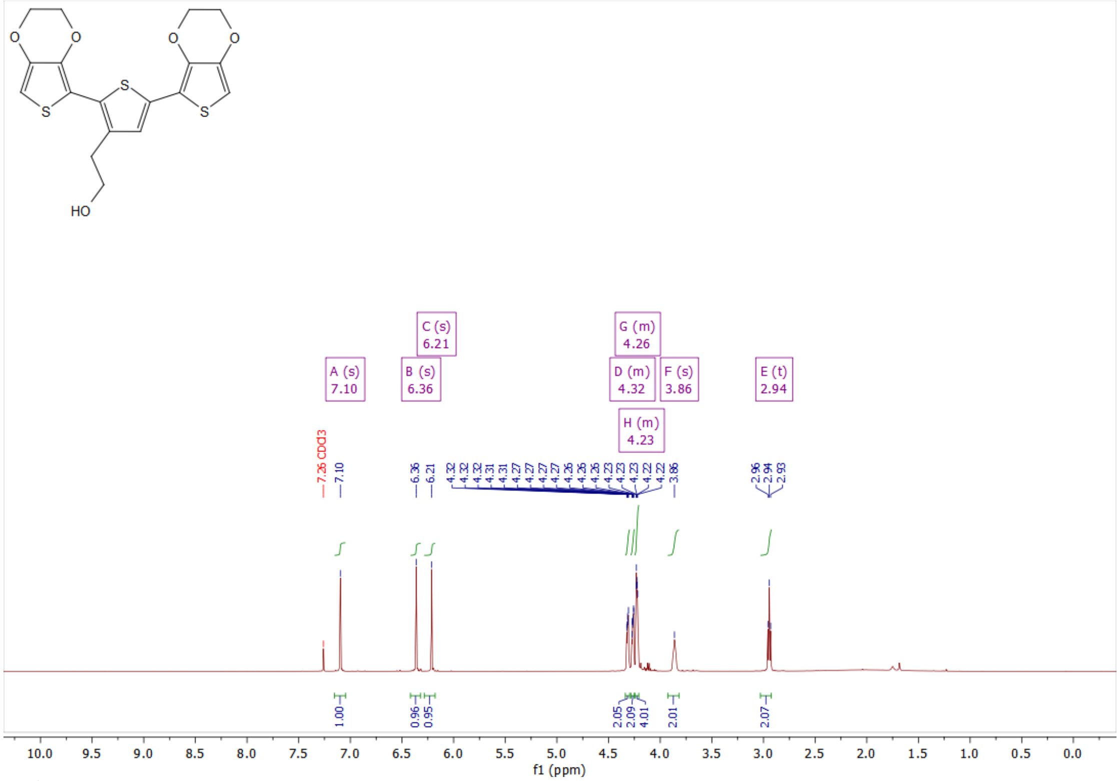

Fig S1. ^{1}H-NMR (chloroform-*d*) spectrum of 2,5-Bis(2,3-dihydrothieno[3,4-*b*]-1,4-dioxin-5-yl)-3-thiopheneethanol (**1**).

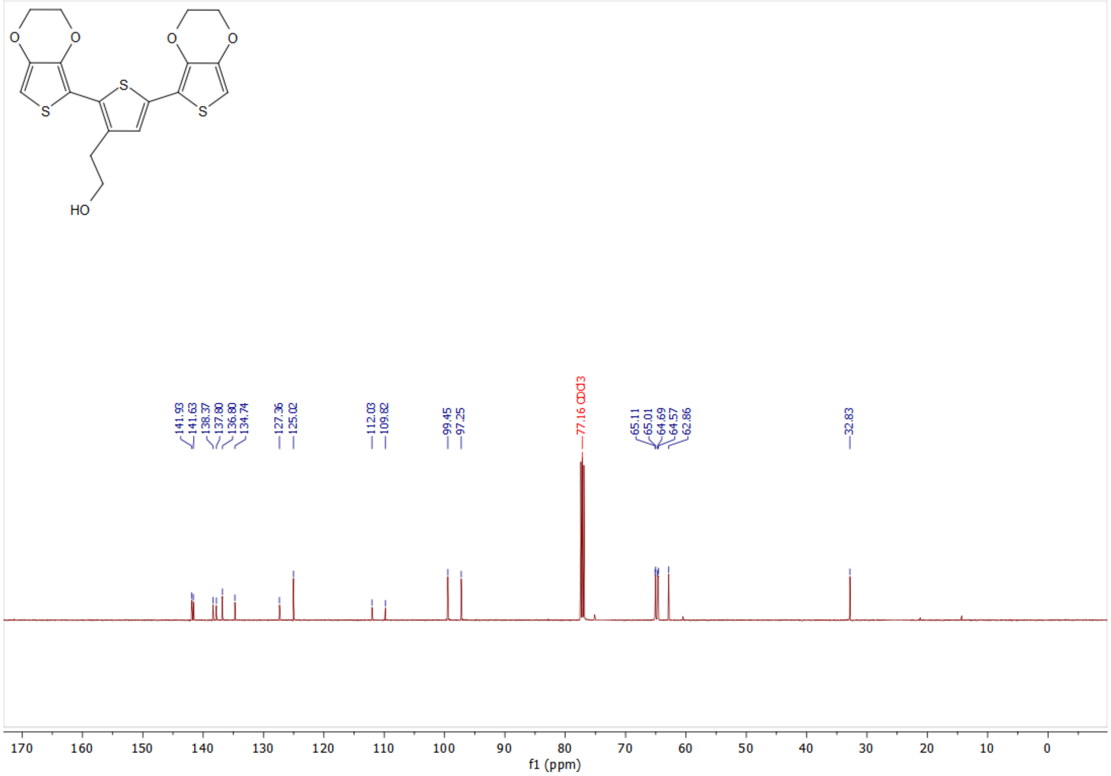

Fig S2. ^{13}C-NMR (chloroform-*d*) spectrum of of 2,5-Bis(2,3-dihydrothieno[3,4-*b*]-1,4-dioxin-5-yl)-3-thiopheneethanol (**1**).

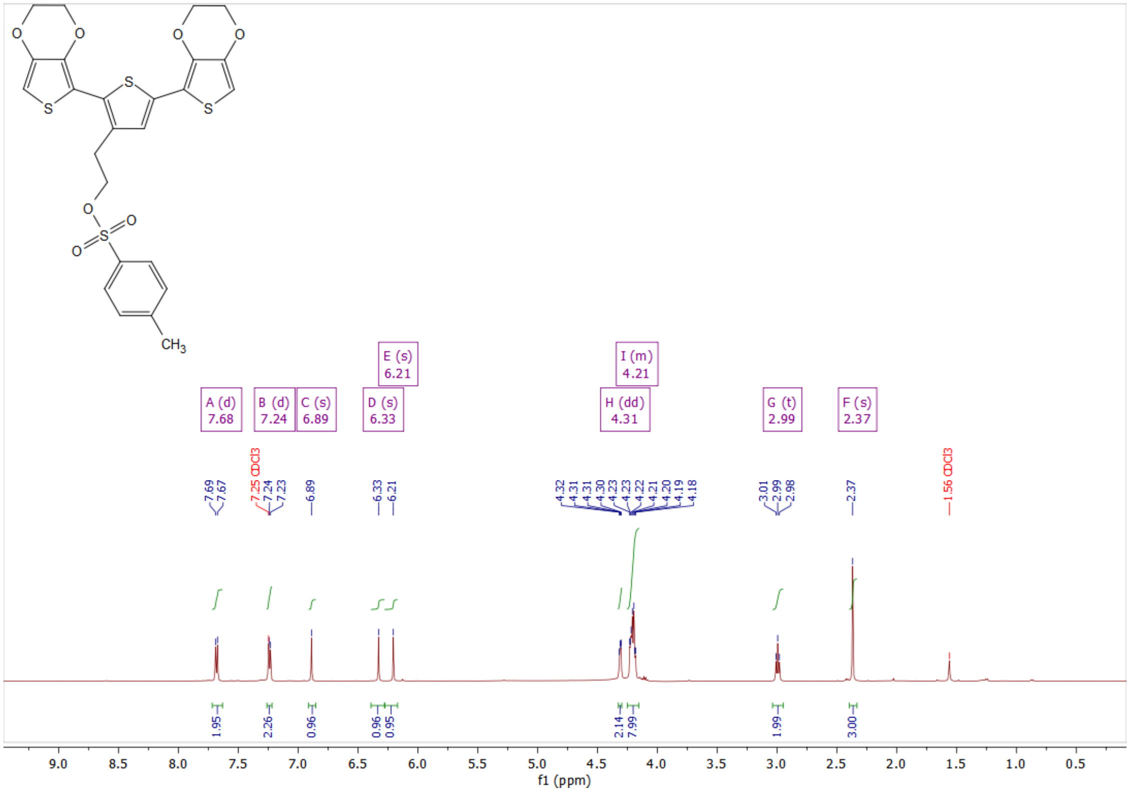

Fig S3. ^{1}H-NMR (chloroform-*d*) spectrum of 2-(2,5-bis(2,3-dihydrothieno[3,4-*b*]-1,4-dioxin-5-yl)thiophene-3-yl)ethyl-4-methylbenzenesulfonate.

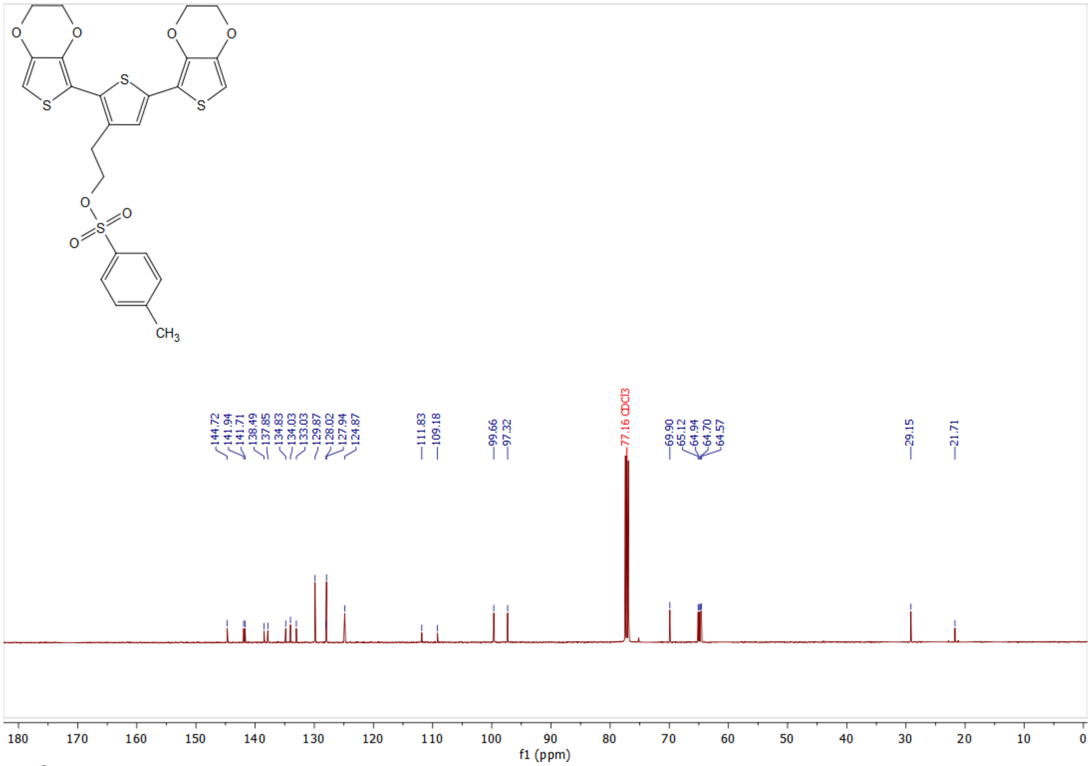

Fig S3. ^{13}C-NMR (chloroform-*d*) spectrum of 2-(2,5-bis(2,3-dihydrothieno[3,4-*b*]-1,4-dioxin-5-yl)thiophene-3-yl)ethyl-4-methylbenzenesulfonate.

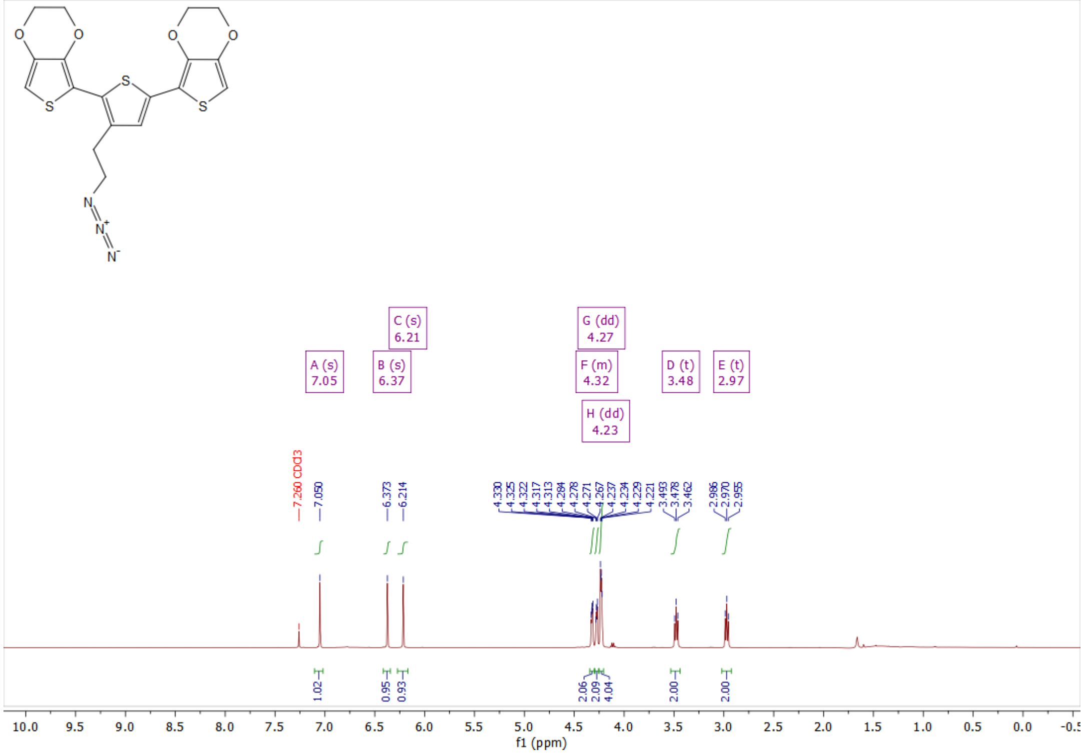

Fig S4. ^{1}H-NMR (chloroform-*d*) spectrum of 3-(2-azidoethyl) 2,5-bis(2,3-dihydrothieno[3,4-*b*]-1,4-dioxin-5-yl)thiophene.

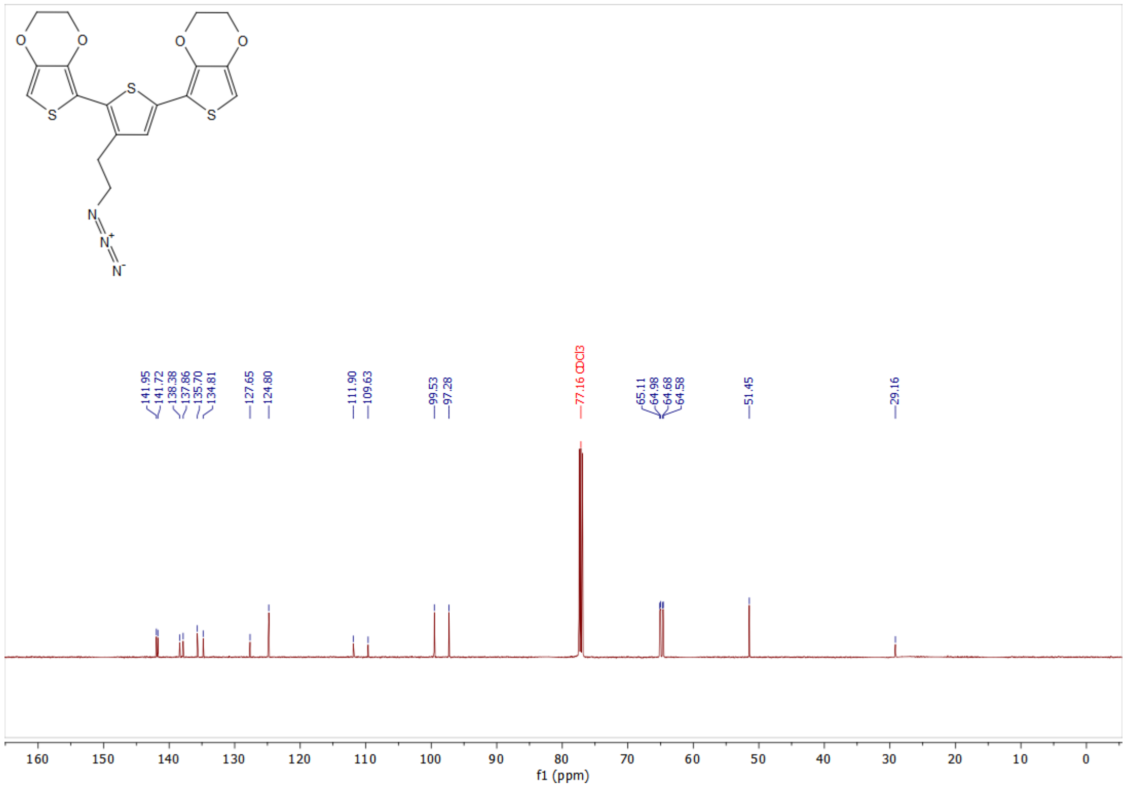

Fig S5. ^{13}C-NMR (chloroform-*d*) spectrum of 3-(2-azidoethyl) 2,5-bis(2,3-dihydrothieno[3,4-*b*]-1,4-dioxin-5-yl)thiophene.

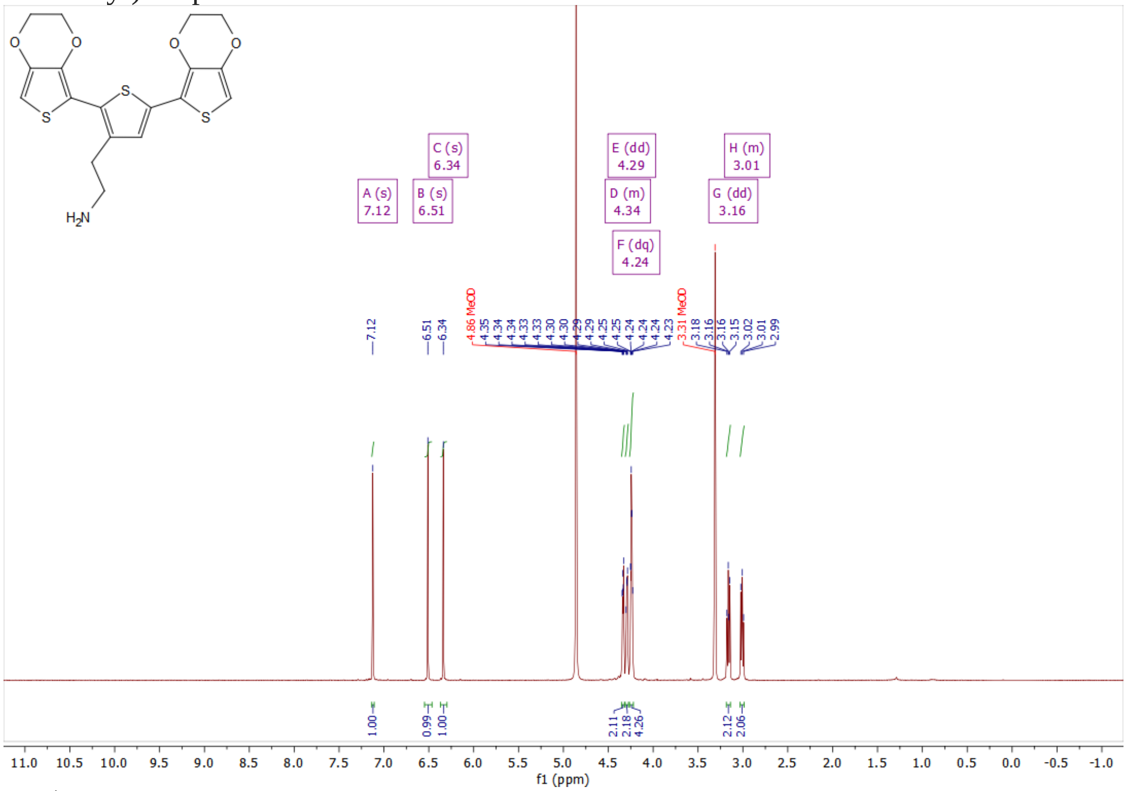

Fig S6. ^{1}H-NMR (methanol-d_4) spectrum of 2-(2,5-bis(2,3- dihydrothieno[3,4-b][1,4]dioxin-5-yl)thiophen-3-yl)ethanamine.

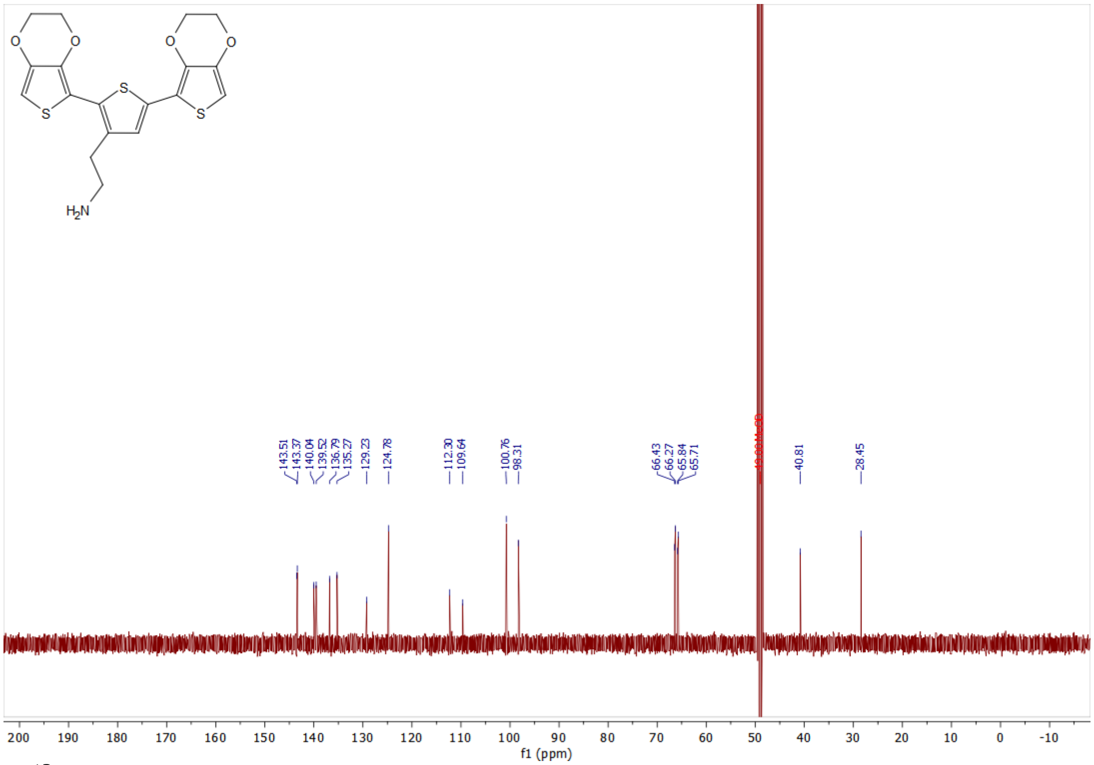

Fig S7. ^{13}C-NMR (methanol-d_4) spectrum of 2-(2,5-bis(2,3- dihydrothieno[3,4-b][1,4]dioxin-5-yl)thiophen-3-yl)ethanamine.

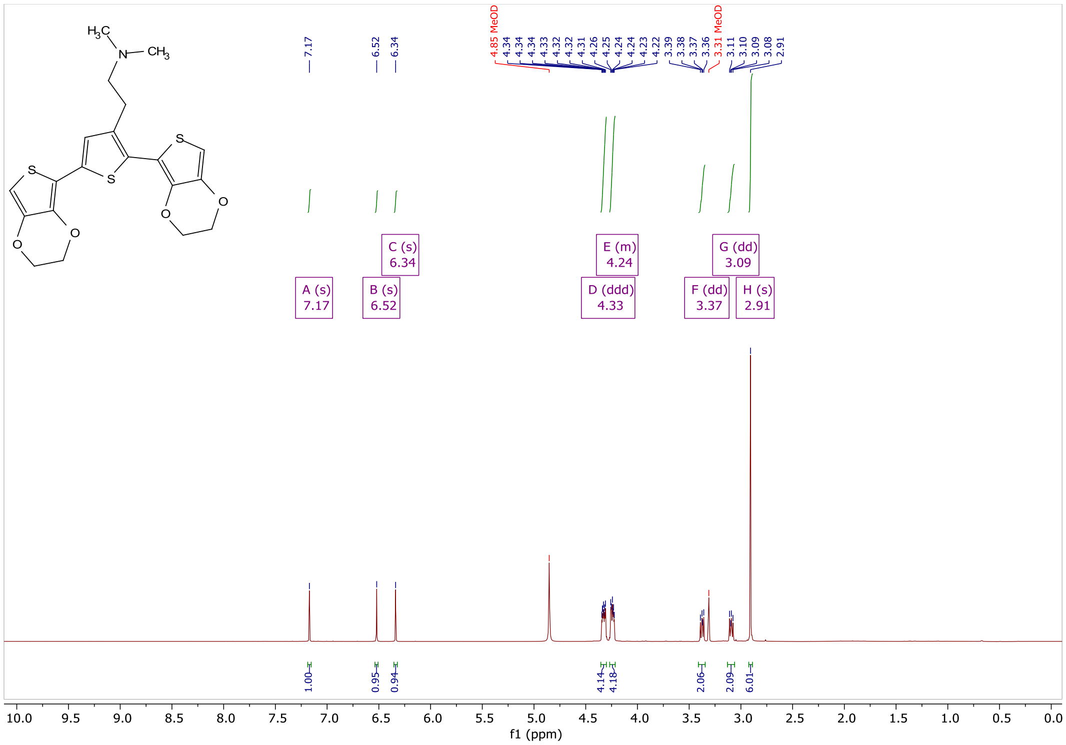

Figure S8. ^{1}H-NMR (Methanol-d_4) spectrum of 2-(2,5-bis(2,3- dihydrothieno[3,4-b][1,4]dioxin-5-yl)thiophen-3-yl)-N,N-dimethylethanamine (**5**).

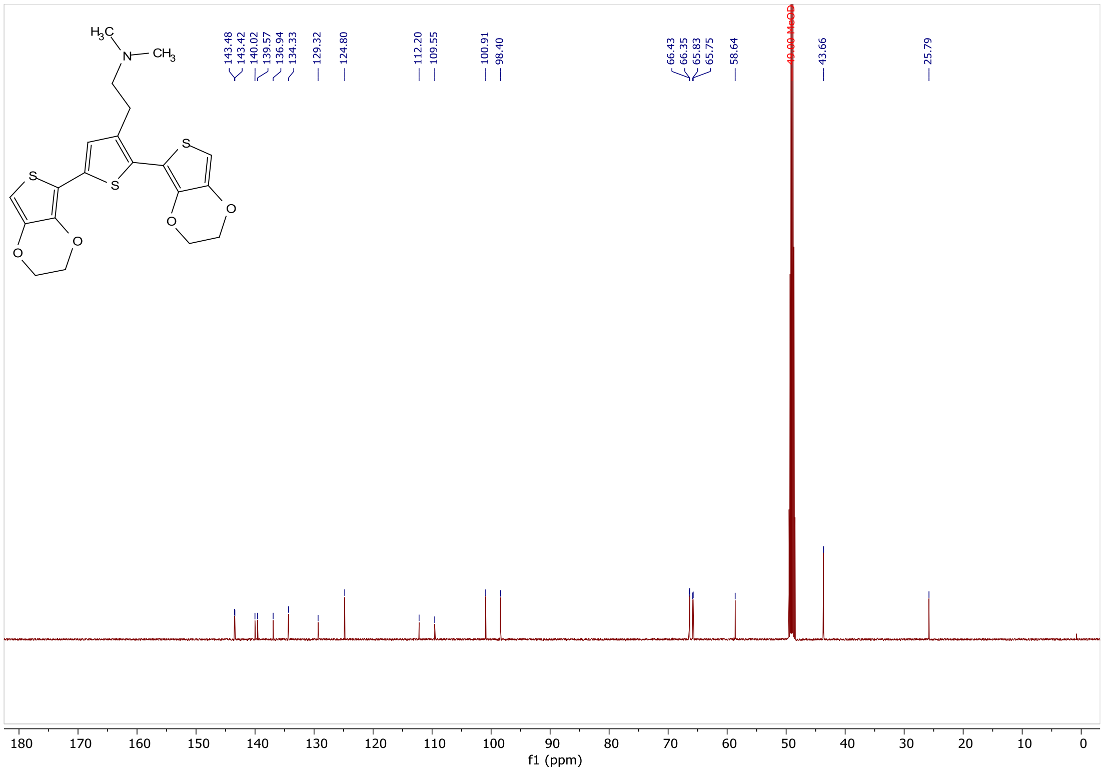

Figure S9. ^{13}C-NMR (Methanol-d_4) spectrum of 2-(2,5-bis(2,3- dihydrothieno[3,4-b][1,4]dioxin-5-yl)thiophen-3-yl)-N,N-dimethylethanamine (**5**).

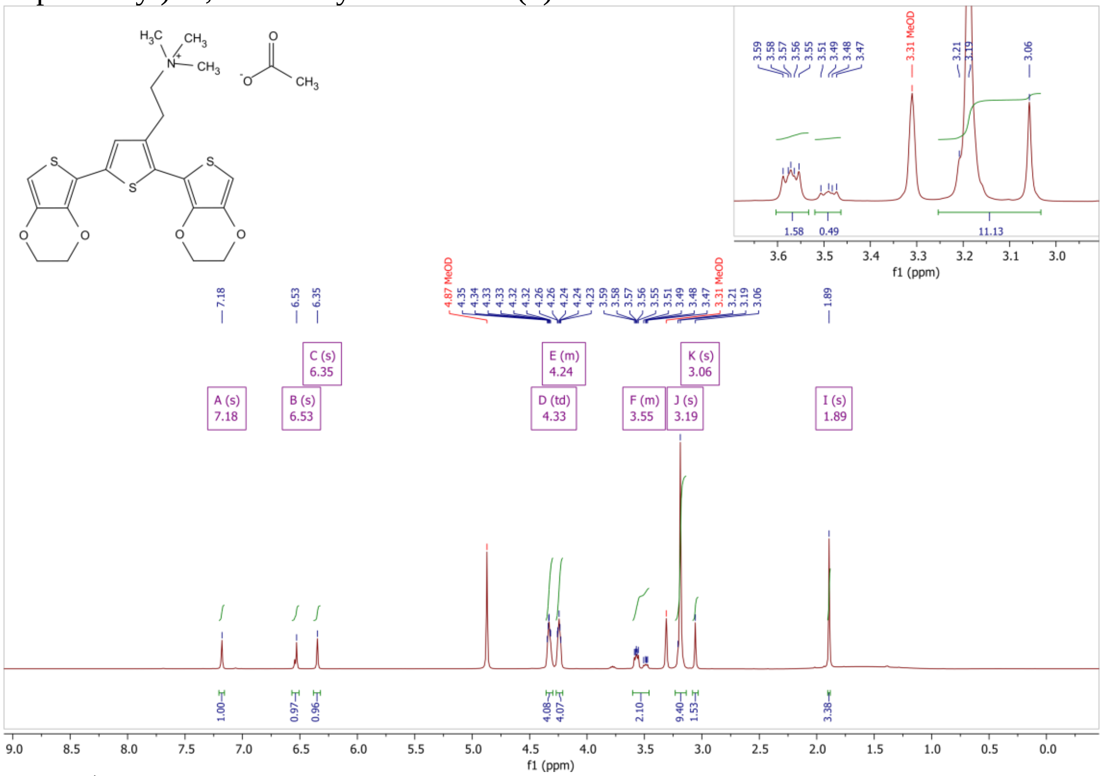

Figure S10. ^{1}H-NMR (Methanol-d_4) spectrum of 2-(2,5-bis(2,3- dihydrothieno[3,4-b][1,4]dioxin-5-yl)thiophen-3-yl)-N,N,N-trimethylethanaminium acetate (**ETE-TMEA**) (**6**).

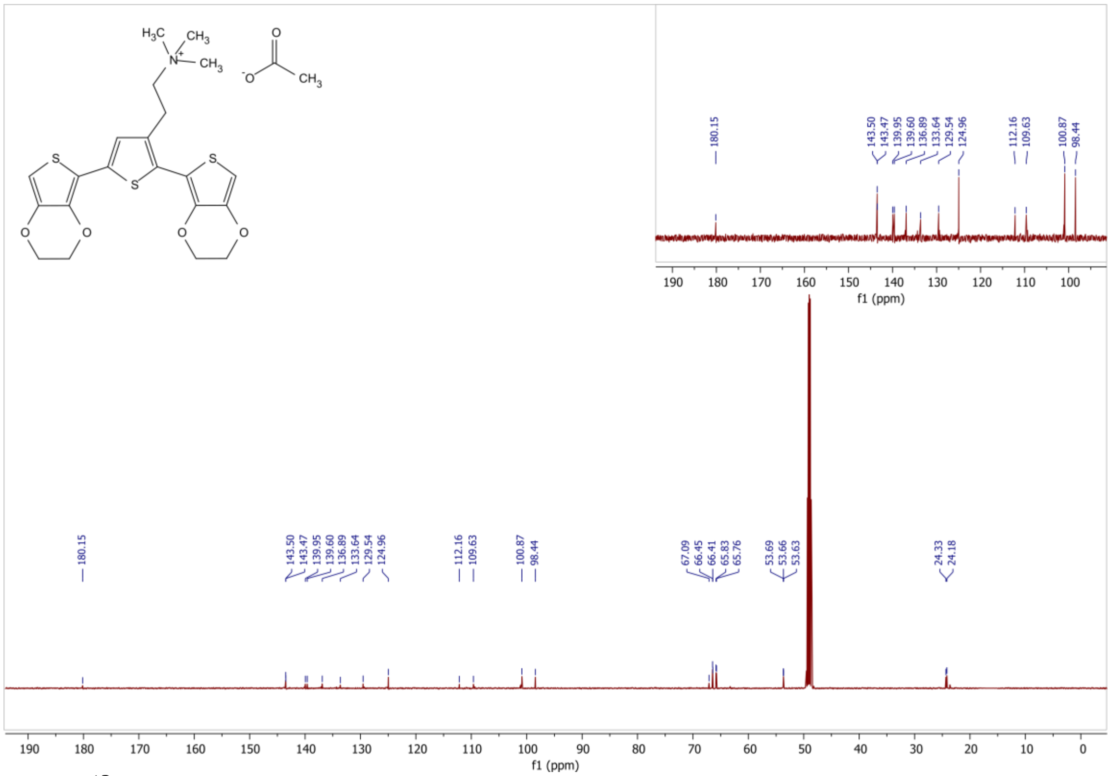

Figure S11. ^{13}C-NMR (Methanol-d_4) spectrum of 2-(2,5-bis(2,3- dihydrothieno[3,4-b][1,4]dioxin-5-yl)thiophen-3-yl)-N,N,N-trimethylethanaminium acetate (**ETE-TMEA**) (**6**).

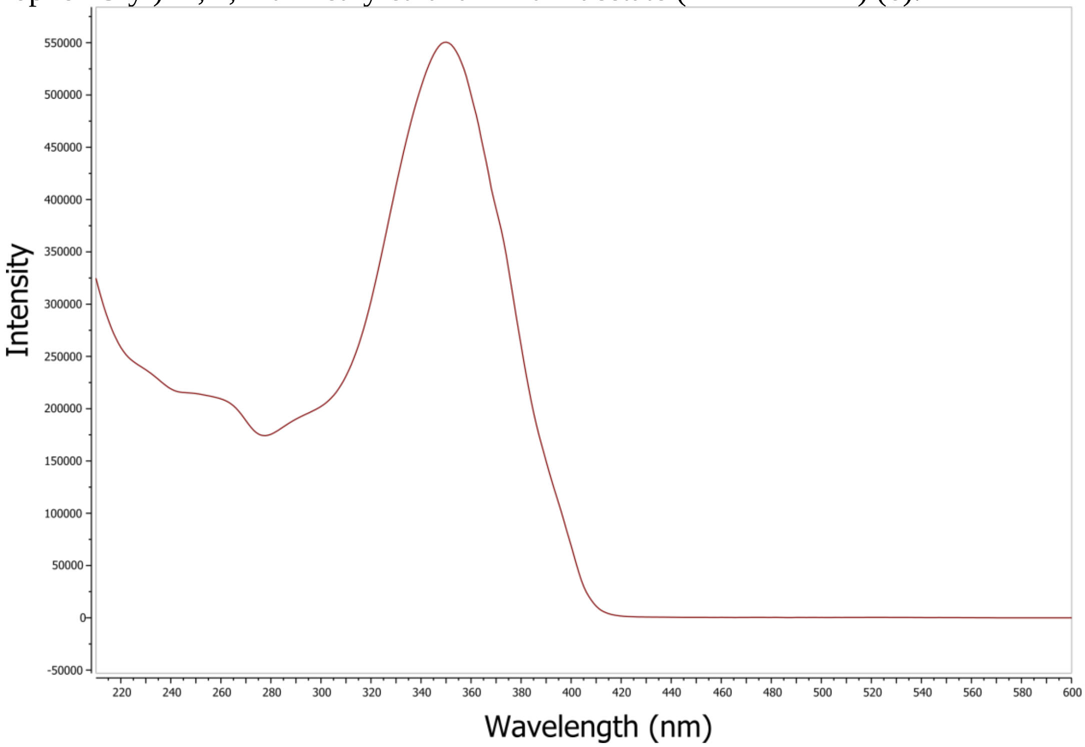

Figure S12. UV-Vis spectrum of 2-(2,5-bis(2,3- dihydrothieno[3,4-b][1,4]dioxin-5-yl)thiophen-3-yl)-N,N,N-trimethylethanaminium acetate (**ETE-TMEA**) (**6**).

Additional spectroscopy data

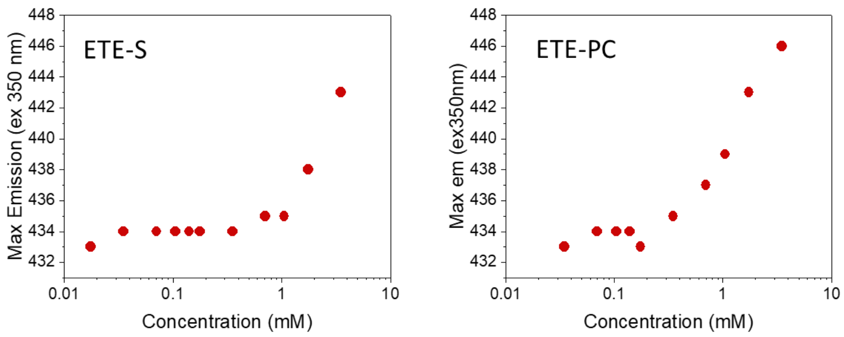

Figure S13. Shift of the emission peaks with concentration (excitation 350 nm)

Dynamic Light Scattering (DLS)

DLS measurements of monomer solutions at concentrations of 3.5 mM and 1 mM in deionised water were performed using Zetasizer Nano ZS90 (Malvern Panalytical) fitted with a 4 mW 632.8 nm laser. UV-transparent disposable polystyrene cuvette covered with cap (Sarstedt) containing 100 µL of the sample was loaded into the instrument and size measurement was conducted at 22 °C by selecting material as protein, dispersant as water, equilibration time of 60 s, and scattering detector angle of 90° in the Zetasizer Software v. 8.02. Average size distribution was obtained using data from 3 measurements per sample, set for an automatically determined number of runs at the rate of 10 s per run, and processed using general purpose analysis model.

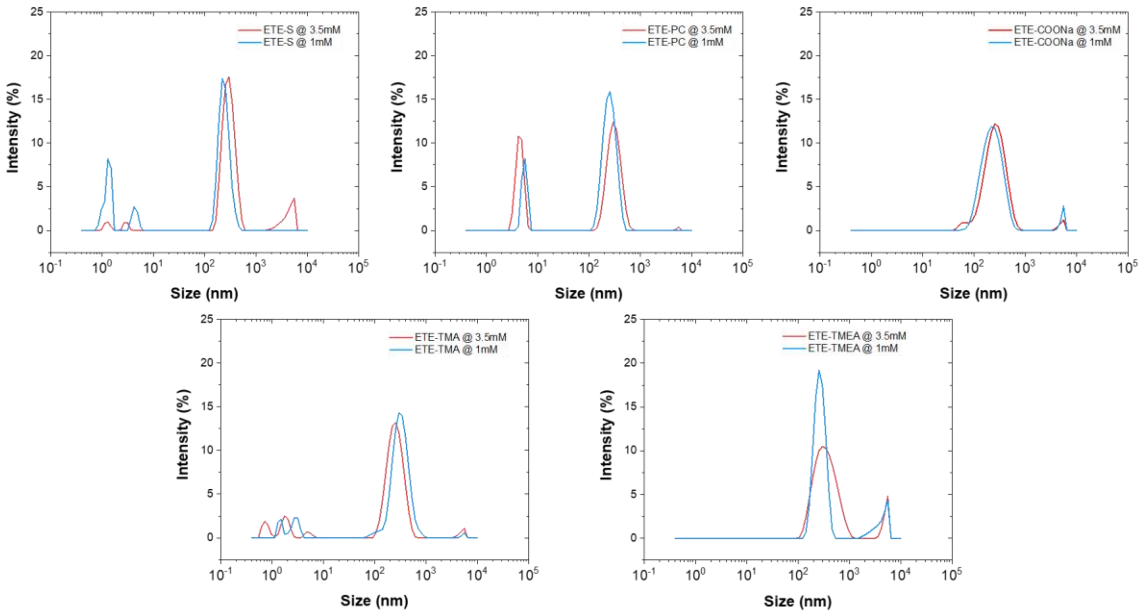

Figure S14. Dynamic light scattering of the five ETEs compounds at high concentrations (3.5 mM and 1 mM) in water.

Additional Modelling data

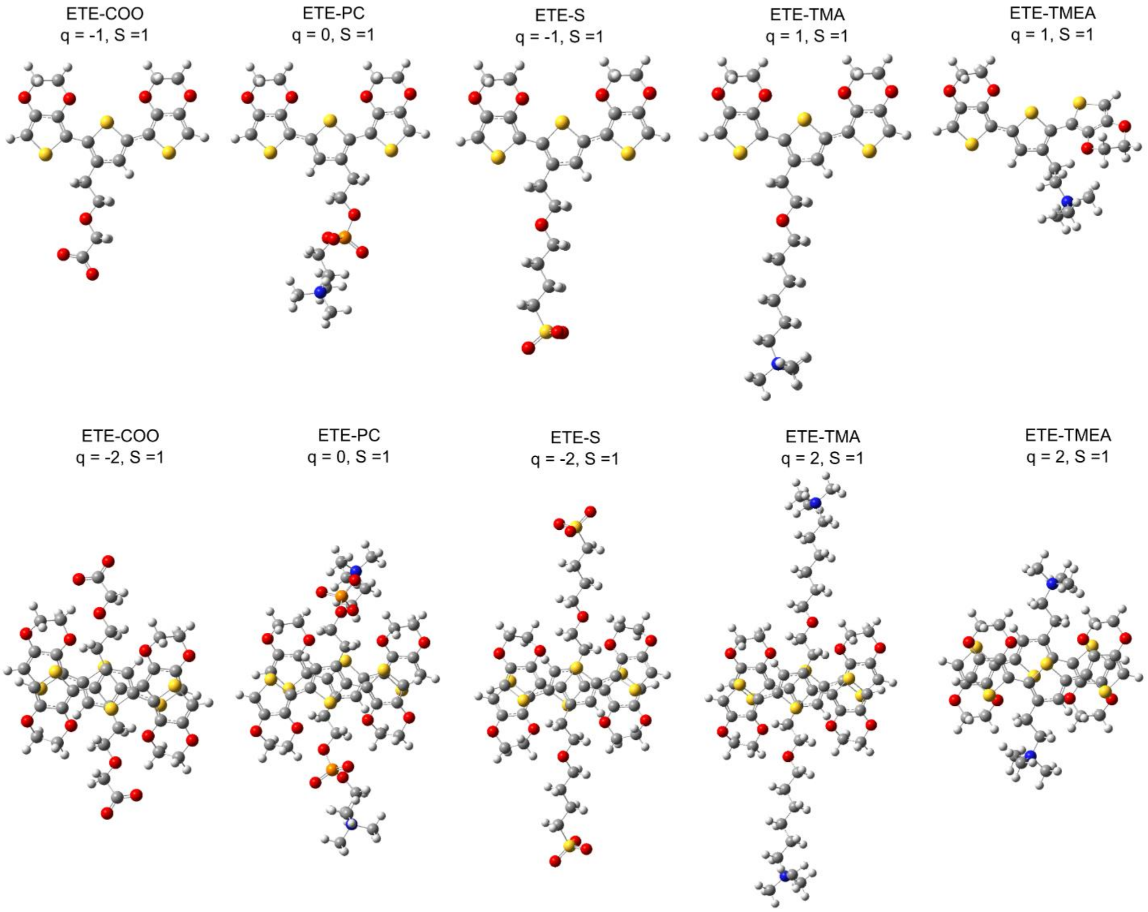

Figure S15. Optimized ground state geometries of monomers and dimers of all five ETE-based compounds.

Table S1

Absorption properties of all compounds using TD-DFT/CAM-B3LYP/6-31G(d)

Species	Transition	λ, nm	f	Assignment
ETE-COO, q=-1, S=1<br>Monomer	S_0-S_1	356.77	0.8730	121 ->122 0.69446
ETE-COO, q=-2, S=1<br>Dimer	S_0-S_1	371.30	0.0002	241 ->244 -0.28005<br>242 ->243 0.63491
ETE-PC, q=0, S=1<br>Monomer	S_0-S_1	356.45	0.8645	150 ->151 0.69441
ETE-PC, q=0, S=1<br>Dimer	S_0-S_1	370.00	0.0204	299 -> 301 -0.20677<br>299 -> 302 -0.29098<br>300 -> 301 0.56264<br>300 -> 302 -0.19419
ETE-S, q=-1, S=1<br>Monomer	S_0-S_1	356.59	0.8694	142 ->143 0.69444
ETE-S, q=-1, S=1<br>Dimer	S_0-S_1	369.97	0.0005	283 -> 286 -0.28864<br>284 -> 285 0.62842
ETE-TMA, q=1, S=1<br>Monomer	S_0-S_1	356.66	0.8687	146 ->147 0.69444
ETE- TMA, q=2, S=1<br>Dimer	S_0-S_1	370.17	0.0005	291 -> 294 -0.28808<br>292 -> 293 0.62896
ETE- TMEA, q=1, S=1<br>Monomer	S_0-S_1	324.98	0.8312	118 ->119 0.69142
ETE- TMEA, q=2, S=1<br>Dimer	S_0-S_1	375.18	0.0001	235 ->238 0.31995<br>236 ->237 0.61631

Table S2

Emission properties of all compounds using TD-DFT/CAM-B3LYP/6-31G(d)

Species	Transition	λ, nm	f	Assignment
ETE-COO, q=-1, S=1<br>Monomer	S_1-S_0	481.02	1.1469	121 ->122 0.69958
ETE-COO, q=-2, S=1<br>Dimer	S_1-S_0	509.99	0.0067	241 ->244 -0.11825<br>242 ->243 -0.68903
ETE-PC, q=0, S=1<br>Monomer	S_1-S_0	480.95	1.1467	150 ->151 0.69958
ETE-PC, q=0, S=1<br>Dimer	S_1-S_0	510.81	0.0129	299 -> 302 0.11426<br>300 -> 301 0.68856
ETE-S, q=-1, S=1<br>Monomer	S_1-S_0	480.81	1.1462	142 ->143 0.69958
ETE-S, q=-2, S=1<br>Dimer	S_1-S_0	515.26	0.0166	283 -> 286 0.11321<br>284 -> 285 -0.69002
ETE-TMA, q=1, S=1<br>Monomer	S_1-S_0	480.76	1.1460	146 ->147 0.69957
ETE- TMA, q=2, S=1<br>Dimer	S_1-S_0	512.15	0.0015	291 -> 294 0.11567<br>292 -> 293 0.68971
ETE- TMEA, q=1, S=1<br>Monomer	S_1-S_0	468.32	1.1000	118 ->119 0.69822
ETE- TMEA, q=2, S=1<br>Dimer	S_1-S_0	519.53	0.0000	235 ->238 -0.11453<br>236 ->237 -0.69049

Table S3

Peaks position in radial distributions functions

Species	The first peak, nm	The second peak, nm	The third peak, nm
ETE-COO	0.42	0.75	
ETE-S	0.45	0.75	1.09
ETE-PC	0.4	0.73	
ETE-TMA	0.41	0.74	
ETE- TMEA	0.38	0.65	

References

1. Gerasimov, J. Y.; Halder, A.; Mousa, A. H.; Ghosh, S.; Harikesh, P. C.; Abrahamsson, T.; Bliman, D.; Strandberg, J.; Massetti, M.; Zozoulenko, I. et al., Rational Materials Design for in Operando Electropolymerization of Evolvable Organic Electrochemical Transistors. *Adv. Funct. Mater.* **2022**, *32*, 2202292.

2. Gerasimov, J. Y.; Zhao, D.; Sultana, A.; Abrahamsson, T.; Han, S.; Bliman, D.; Tu, D.; Simon, D. T.; Olsson, R.; Crispin, X. et al., A Biomimetic Evolvable Organic Electrochemical Transistor. *Adv. Electron. Mater.* **2021**, *7*, 2001126.

3. Strakosas, X.; Biesmans, H.; Abrahamsson, T.; Hellman, K.; Ejneby, M. S.; Donahue, M. J.; Ekström, P.; Ek, F.; Savvakis, M.; Hjort, M.; Bliman, D. et al., Metabolite-Induced in Vivo Fabrication of Substrate-Free Organic Bioelectronics. *Science* **2023**, *379*, 795-802

4. Gottlieb, H. E.; Kotlyar, V.; Nudelman, A., Nmr Chemical Shifts of Common Laboratory Solvents as Trace Impurities. *J. Org. Chem.* **1997,** *62*, 7512-7515.
